# IL-22 Restrains Tapeworm-Mediated Protection against Experimental Colitis via Regulation of IL-25 Expression

**DOI:** 10.1371/journal.ppat.1005481

**Published:** 2016-04-07

**Authors:** José L. Reyes, Maria R. Fernando, Fernando Lopes, Gabriella Leung, Nicole L. Mancini, Chelsea E. Matisz, Arthur Wang, Derek M. McKay

**Affiliations:** Gastrointestinal Research Group, Department of Physiology and Pharmacology, Calvin, Joan and Phoebe Snyder Institute for Chronic Diseases, Cumming School of Medicine, University of Calgary, Calgary, Alberta, Canada; University of California San Francisco, UNITED STATES

## Abstract

Interleukin (IL)-22, an immune cell-derived cytokine whose receptor expression is restricted to non-immune cells (e.g. epithelial cells), can be anti-inflammatory and pro-inflammatory. Mice infected with the tapeworm *Hymenolepis diminuta* are protected from dinitrobenzene sulphonic acid (DNBS)-induced colitis. Here we assessed expulsion of *H*. *diminuta*, the concomitant immune response and the outcome of DNBS-induced colitis in wild-type (WT) and IL-22 deficient mice (IL-22^-/-^) ± infection. Interleukin-22^-/-^ mice had a mildly impaired ability to expel the worm and this correlated with reduced or delayed induction of TH2 immunity as measured by splenic and mesenteric lymph node production of IL-4, IL-5 and IL-13 and intestinal Muc-2 mRNA and goblet cell hyperplasia; in contrast, IL-25 increased in the small intestine of IL-22^-/-^ mice 8 and 12 days post-infection compared to WT mice. *In vitro* experiments revealed that *H*. *diminuta* directly evoked epithelial production of IL-25 that was inhibited by recombinant IL-22. Also, IL-10 and markers of regulatory T cells were increased in IL-22^-/-^ mice that displayed less DNBS (3 mg, ir. 72h)-induced colitis. Wild-type mice infected with *H*. *diminuta* were protected from colitis, as were infected IL-22^-/-^ mice and the latter to a degree that they were almost indistinguishable from control, non-DNBS treated mice. Finally, treatment with anti-IL-25 antibodies exaggerated DNBS-induced colitis in IL-22^-/-^ mice and blocked the anti-colitic effect of infection with *H*. *diminuta*. Thus, IL-22 is identified as an endogenous brake on helminth-elicited TH2 immunity, reducing the efficacy of expulsion of *H*. *diminuta* and limiting the effectiveness of the anti-colitic events mobilized following infection with *H*. *diminuta* in a non-permissive host.

## Author Summary

Interleukin (IL)-22, produced by innate and adaptive immune cells, plays a complex role in immunity; under specific conditions, targeting this cytokine could treat inflammatory diseases. The hygiene hypothesis suggests infection with helminth parasites could ameliorate inflammation. Here we show that IL-22 is required to activate early events (i.e. type 2 cytokines and mucin expression) in the response to the non-invasive cestode *Hymenolepis diminuta*. Strikingly, expression of regulatory factors (IL-10, IL-25, Foxp3), which arise following *H*. *diminuta* infection, were either enhanced or sustained in IL-22^-/-^ mice, uncovering a novel role for IL-22 as a brake for these regulatory events following infection with this parasitic helminth. Moreover, DNBS-induced colitis was significantly less severe in IL-22^-/-^ compared to wild-type mice: IL-22^-/-^ mice infected with *H*. *dimunta* 8-days prior to the induction of colitis had negligible disease. Immunoneutralization of IL-25 exaggerated DNBS-induced colitis in the IL-22^-/-^ mice and ablated the anti-colitic effect of infection with *H*. *diminta*. Thus, while immune events in the early response to infection with *H*. *diminuta* are delayed in IL-22^-/-^ mice (as is worm expulsion), the compensatory enhancement of IL-25 (and other immunoregulatory elements (e.g. IL-10)) provide resistance to colitis and also promote the anti-colitic effect driven as a consequence of the response to infection with *H*. *diminuta*. The data confirm the complex role of IL-22 in intestinal immunity.

## Introduction

Interleukin (IL)-22, a member of the IL-10 family, is produced predominantly by innate (NK cells (NK_22_), γδ T cells, innate lymphoid cells type 3 (ILC3s) and adaptive (CD4^+^ Th22 and Th17, CD8^+^ T cells) immune cells: a non-immune source of IL-22 has not been described. The heterodimeric IL-22 receptor consists of the IL-10R2 subunit and the unique IL-22R1 subunit, and is restricted to non-hematopoetic cells (e.g. hepatocytes and epithelium of the gastrointestinal tract) [1]. Thus, IL-22 is an immune cell-derived mediator that acts exclusively on non-immune cells and as such is an attractive target for therapeutic intervention [2, 3].

Data from studies of the gastrointestinal tract suggest that the role of IL-22 is contextual, with beneficial or detrimental affects depending on the nature of the disease or immune activity being assessed. For example, IL-22^-/-^ mice are more susceptible to colitis induced by dextran sodium sulfate (DSS) [4] and retinoic acid suppression of DSS-induced colitis was associated with increased IL-22 [5]. Similarly, local delivery of the IL-22 gene attenuated the spontaneous colitis that develops in T cell receptor (TCR)-α knockout (KO) mice [6] and that evoked by transfer of naïve CD45RB^hi^ T cells into RAG2^-/-^ mice [4]. However, IL-22, mobilized by IL-23, was implicated in the exaggeration of murine colitis induced by anti-CD40 activation in RAG1^-/-^ mice [7]. Fewer IL-22^+^ cells have been described in inflamed tissue from patients with ulcerative colitis compared to healthy individuals [8]. In contrast, ILC3 from patients with mild-moderate ulcerative colitis were reported to have increased IL-22 production [9].

This duality of IL-22 function extends beyond the gut. Interleukin-22 can promote hepatocyte survival in acute mouse models of liver damage [10], while IL-22 recruitment of Th17 cells has been implicated in chronic liver inflammation of hepatitis B-infected individuals [11]. Pro- and anti-inflammatory roles have been described for IL-22 in murine models of arthritis [12]; for example, IL-22 was implicated in the enhancement or suppression of collagen-induced arthritis in mice co-treated with the parasitic nematode-derived molecule, ES-62 [13].

The role of IL-22 following infection is equally diverse, where it has been shown to protect mice from infection with *Citrobacter rodentium* and *Salmonella enterica* [14], but appears not to affect the outcome of infection with *Mycobacterium avium* [15]; susceptibility to *Salmonella* has been reported [16]. The route of pathogen entry into the body can be important, IL-22 acting downstream of IL-23, promoted resistance against intragastrically or intravenously delivered *Candida albicans* [17], but played no role in the response to cutaneous *C*. *albicans* [18]. Two independent studies demonstrated roles for IL-22 in the intestinal pathophysiology associated with infection with *Toxoplasma gondii* [15, 19]. With respect to infection with helminth parasites, Wilson et. al. found no role for IL-22 in the murine response to *Schistosoma mansoni* [15], whereas goblet cell hyperplasia and mucin secretion, a key effector in the gut, was driven by IL-22 following infection with nematodes [20]. Increased IL-22 has been demonstrated in individuals with established hookworm infection although its function was not defined [21]. A report of self-infection with the nematode parasite *Trichuris trichiura* to treat ulcerative colitis documented increased numbers of CD4^+^IL22^+^ cells [22].

Infection with the rat tapeworm, *Hymenolepis diminuta*, protects mice from colitis induced by intra-rectal (i.r.) instillation of the haptenizing agent, 2,4-dinitrobenzene sulphonic acid (DNBS) [23]. Given the pivotal role that IL-22 can play in immune-stromal cell communication and the disparite data on this cytokine in the response to infection (and general lack of data in relation to helminths) and regulation of inflammation, the current study assessed the impact of the absence of IL-22 in (1) the expulsion of *H*. *diminuta* from its non-permissive mouse host and the concomitant immune response, and (2) whether the anti-colitic effect of infection with *H*. *diminuta* was modified.

## Results and Discussion

### IL-22^-/-^ mice display defective expulsion of *H*. *diminuta* and reduced early TH2 response

**Fig 1:**
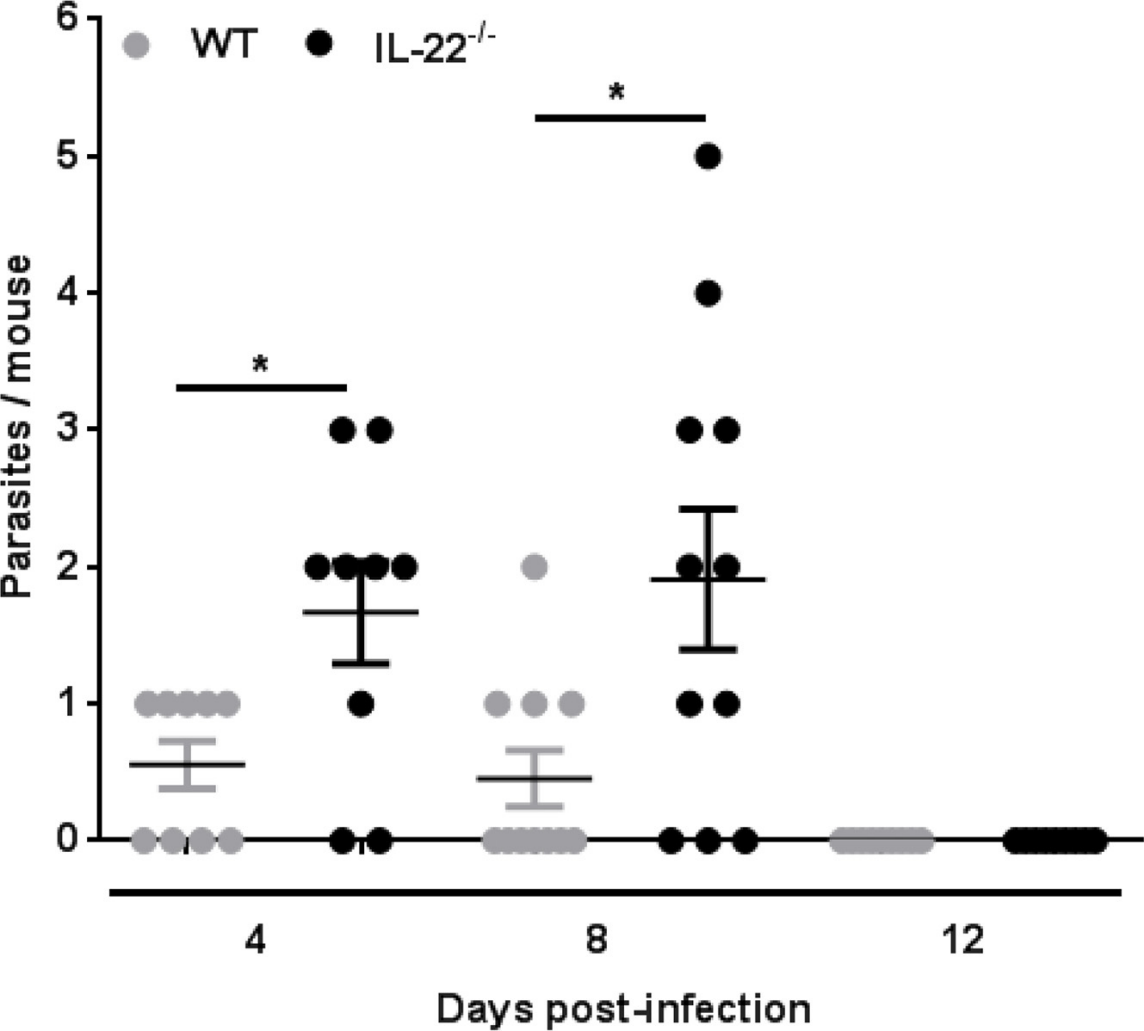
Absence of IL-22 alters the expulsion kinetics of *H*. *diminuta* from mice. Wild-type (WT; gray symbols) and IL-22^-/-^ (black symbols) mice were orally infected with 5 *H*. *diminuta* cysticrecoids and at the indicated time-points were euthanized, the small intestine removed, flushed with PBS and retrieved parasites counted (lines indicate mean ± SEM; n = 6–9 at each point from 3 independent experiments; *p <0.05 by student’s *t* test).

The role of IL-22 in modifying the host response to infection with helminth parasites appears to be determined by the nature of the infection. For example, worm burden and granuloma size is not different in schistosoma-infected WT and IL-22^-/-^ mice [15], whereas IL-22 was important in the goblet cell hyperplasia and mucin secretion response following infection with the intestinal nematodes, *Trichuris muris* and *Nippostrongylus brasiliensis* [20]. The tapeworm *H*. *diminuta* is unique amongst helminths that infect the intestine as it does negligible, if any, damage to the host: it lacks a tissue migratory phase and the absence of hooks on the scolex means it is not abrasive. IL-22^-/-^ mice displayed a slight delay in the kinetics of expulsion of *H*. *diminuta*: only 22% (2/9 mice) of infected IL-22^-/-^ mice had expelled *H*. *diminuta* by 8 days post-infection (dpi) compared to 55% (5/9 mice) of WT mice (Fig 1); at this time-point 33% of infected IL-22^-/-^ mice harboured 3 or 4 worms, burdens not observed in WT mice. At 12 dpi, *H*. *diminuta* had been completed expelled from WT and IL-22^-/-^ mice, suggesting that while IL-22 signaling promotes a rapid anti-*H*. *diminuta* response the duration of infection is not prolonged in the absence of this cytokine.

**Fig 2:**
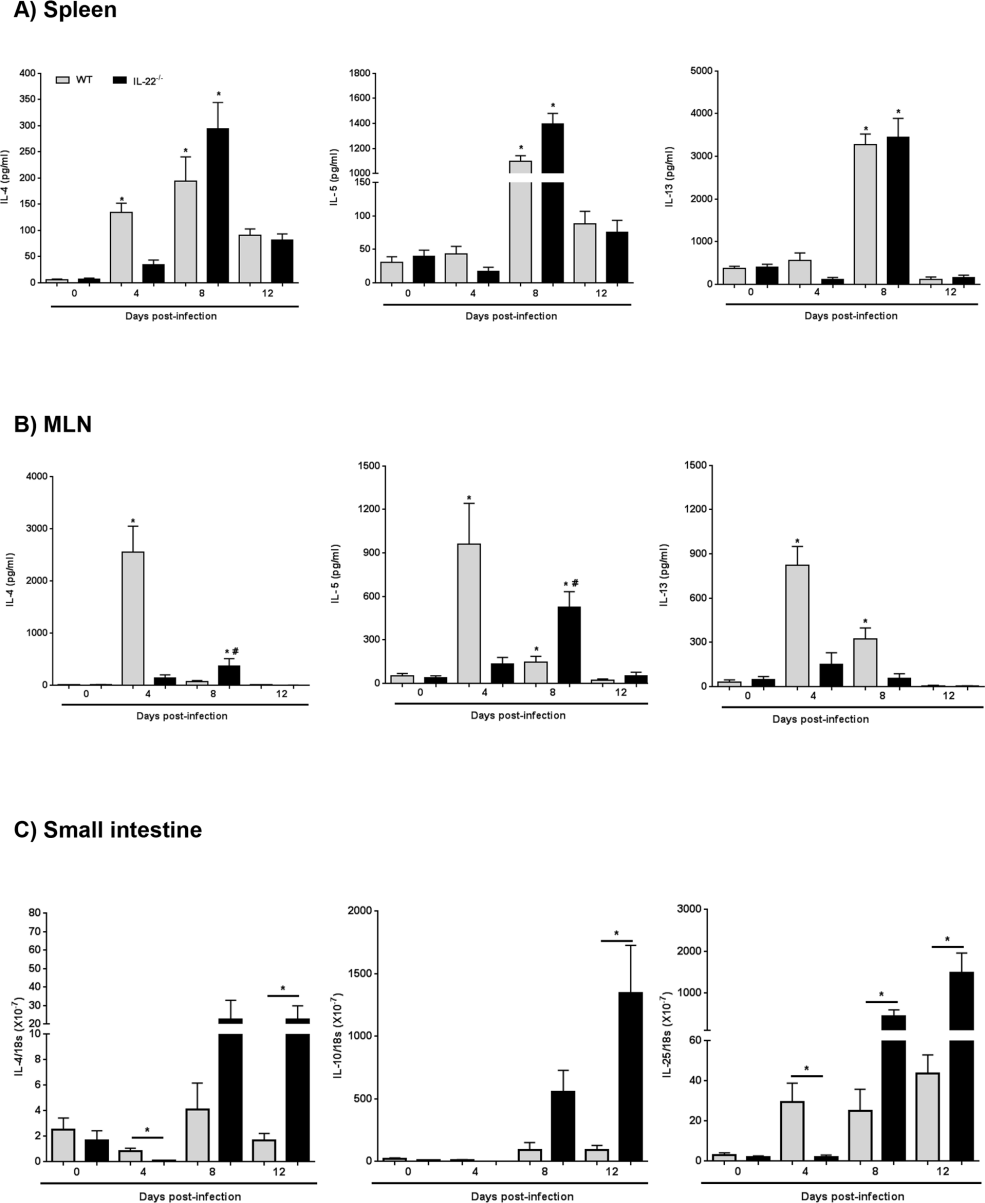
IL-22 absence results in altered TH2 immune responses. Wild-type (WT; gray bars) and IL-22^-/-^ (black bars) mice were infected with 5 *H*. *diminuta* cysticrecoids and at indicated time-points were euthanized and spleen (A), mesenteric lymph node (MLN) (B) and portions of mid-small intestine (C) were excised. Spleen and MLN cell suspensions were generated and incubated for 48 hr with conA (5 μg/ml) and TH2 cytokines quantified by ELISA. Intestinal tissue was assessed by qPCR (data are mean ± SEM; n = 9 from 3 independent experiments; * and #, p <0.05 compared to control and time-matched WT, respectively).

Mobilization of TH2-type cytokines (i.e. IL-4, IL-5 and IL-13) is a hallmark of the immune response following infection with parasitic helminths [24]. Consistent with previous findings [25], mitogen stimulation of splenocytes or mesenteric lymph node (MLN) cells from WT mice resulted in increased IL-4, IL-5 and IL-13 by 4-dpi (Fig 2A and 2B), declining to control levels by 12-dpi. Time-matched analyses revealed reduced levels of the 3 cytokines from MLN and spleen of IL-22^-/-^ mice on day 4-dpi compared to WT mice, that rebounded to match or exceed those of WT mice by day 8-pdi (the exception being IL-13 production by MLN cells) (Fig 2A and 2B). By 12-dpi there were no differences in splenic and MLN-derived IL-4, IL-5 or IL-13 in infected WT and IL-22^-/-^ mice. Measurement of the TH1 cytokine IFN-γ from conA-stimulated splenocytes revealed no differences between WT and IL-22^-/-^ mice over the 12-day infection period (S1 Fig). In addition, qPCR revealed reduced expression of IL-4, IL-10 and IL-25 mRNA in intestinal tissue from infected IL-22^-/-^ mice compared to WT animals at 4-dpi, with a rebound heightened expression in all 3 cyokines by 8-dpi, which unlike the spleen and MLN was extended until 12-dpi (Fig 2C) (end of experiment). This delay in the production of key TH2 effector cytokines parallels the delay in expulsion of *H*. *diminuta* from IL-22^-/-^ mice and the events are likely to be causally linked. These data align with the requirement for IL-25 in the expulsion of nematode parasites from mice [26–28]. Fascinatingly, and in accordance with IL-22’s dual functions [13], the diminished TH2 responses in IL-22^-/-^ *H*. *diminuta*-infected mice suggests an important role for innate immunity early in the response to helminths and additional studies are needed to precisely define this.

**Fig 3:**
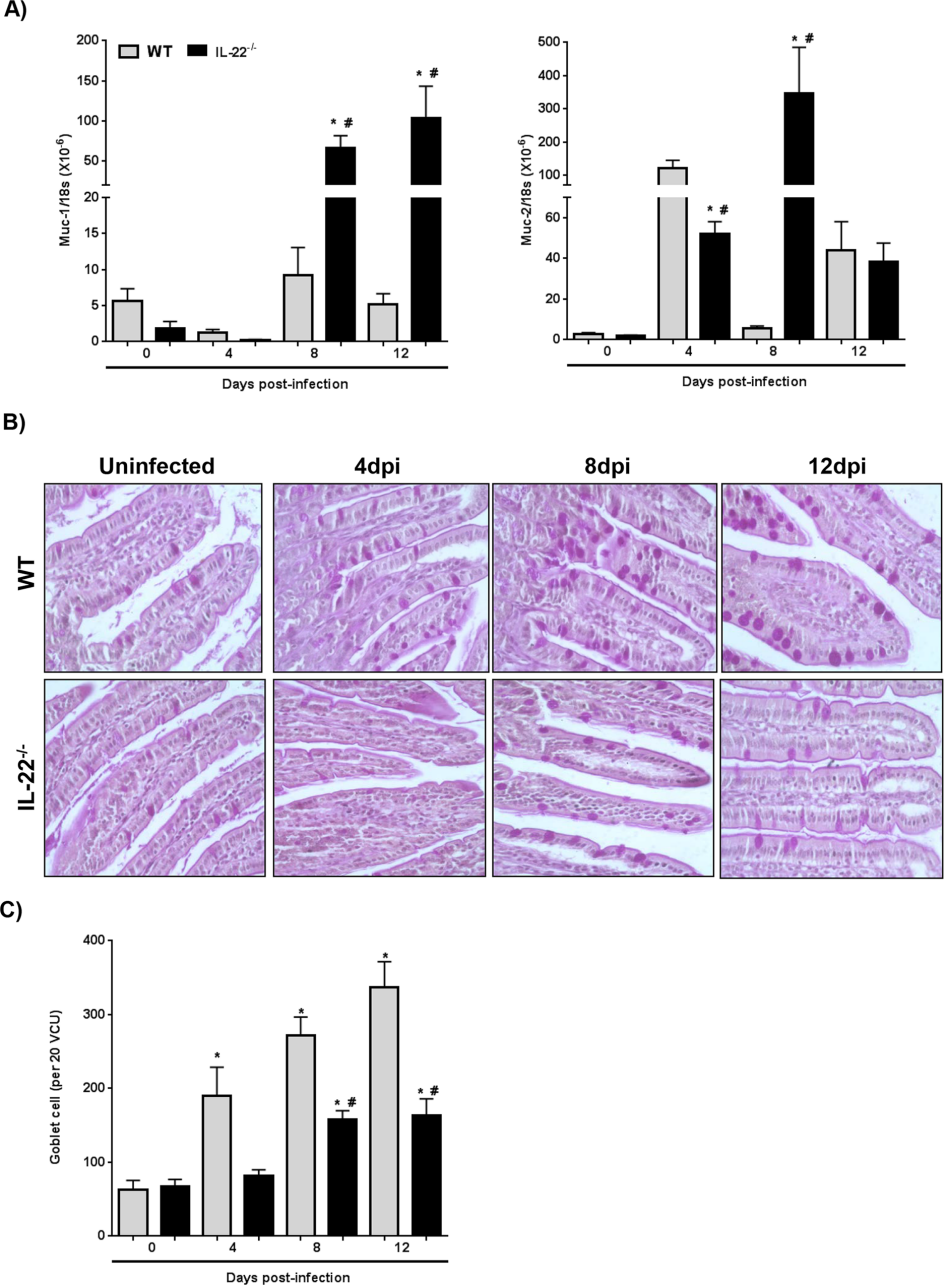
Mice lacking IL-22 display reduced expression of mucin mRNA and fewer goblet cells at early time-points following infection with *H*. *diminuta*. Small intestine from WT (gray bars) and IL-22^-/-^ (black bars) mice was flushed with sterile PBS and mRNA extracted from mid-small intestine and used to analyze Muc-1 and Muc-2 mRNA by qPCR (A) (target gene normalised against RNA *18s* housekeeping gene). Goblet cells were identified by periodic acid-Schiff staining of sections on coded slides (B, C) and enumerated microscopically base on intact villus-crypt units (VCU) (data are mean ± SEM; n = 6–9 from 3 experiments, * and #, p <0.05 compared to control and time-matched WT, respectively).

Interleukin-4 has been implicated in the regulation of goblet cell hyperplasia following infection with helminth parasites [29]. Indeed, mucin synthesis and release are important, often critical, effector responses against enteric helminths [30] and goblet cell hyperplasia follows the kinetics of *H*. *diminuta* expulsion from WT mice [31]. Four dpi mRNA for the secreted mucin, Muc-2, was increased in the small intestine of infected WT and to a lesser extent in IL-22^-/-^ mice: and while Muc-2 mRNA expression declined in the intestine of WT mice, in IL-22^-/-^ mice the elevated Muc-2 expression was maintained at 8-dpi, paralleling the kinetics of *H*. *diminuta* expulsion (Fig 3A). The Muc-1 gene encodes a transmembrane bound mucin; little is known of its function [32]. Muc-1 mRNA was significantly upregulated in *H*. *diminuta*-infected IL-22^-/-^ mice at 8- and 12-dpi and it is tempting to speculate that this might compensate for the reduced Muc-2 signal at 4-dpi in these mice (Fig 3A). Rats, the natural definitive host for *H*. *diminuta*, infected with 5 cysticercoids show no increase in Muc-2 mRNA, whereas a 50 cysticercoid oral inoculum resulted in increased Muc-2 mRNA, and ≤15 worms established in the gut [33].

Histochemical staining revealed increased numbers of mucus-containing goblet cells in the small intestine of *H*. *diminuta*-infected WT mice (Fig 3B) [31]; however, intestine from infected IL-22^-/-^ mice displayed no significant increase in goblet cells at 4-dpi (Fig 3B). The reduced Muc-2 expression and parallel changes in goblet cell numbers in IL-22^-/-^ mice could contribute to the increased worm burden observed at 8-dpi, while maintenance of the Muc-2 signal and sustained goblet cell numbers (Fig 3C) may allow for these mice to catch-up with WT animals, fully expelling *H*. *diminuta* by 12-dpi. However, neither Muc2 mRNA nor goblet cell numbers are substantially increased beyond WT levels at 12-dpi, despite increased IL-4 and IL-25 mRNA in the small intestine, suggesting that once the parasite has been eradicated (see Fig 1), regulatory mechanisms come into play to dampen a mucus/goblet cell response. Interleukin-22 has been implicated in the barrier function of the gut, especially the secretion of anti-microbial factors and mucin [34], and while this can be a direct effect, the diminutation of IL-4 or IL-13 production in the IL-22^-/-^ mice could contribute to the perturbation of mucin and goblet cell regulation following infection with helminth parasites.

Intestinal mast cell hyperplasia can accompany infection with nematodes [24], but c-Kit immunostaining revealed comparable numbers (and distribution) of mast cells in WT and IL-22^-/-^ mice (S2 Fig). These data suggest a limited, if any, role for mast cells in the current study but an in-depth analysis is required before definitive statements on the role of mast cells (with or without IL-22) in the response to *H*. *diminuta* can be made.

### IL22^-/-^ mice infected with *H*. *diminuta* have delayed but enhanced up-regulation of IL-25, IL-10 and Foxp3

Juxtaposing the facts that the epithelium is a target for IL-22 [1] and epithelium-derived factors are important in shaping the immune response and the outcome of infection [35], the impact of the absence of IL-22 on the mobilization of regulatory immune factors/cells was assessed following infection with *H*. *diminuta*. The observation of increased IL-25 mRNA in the jejunum of IL-22^-/-^ mice at 8- and 12-dpi with *H*. *diminuta* (Fig 2C) suggested that IL-22 serves as a brake on the synthesis of tissue (i.e. epithelial)-derived cytokines elicited in response to infection with helminth parasites. The increase in IL-25 mRNA in the IL-22^-/-^ mice could be due to increased presence of the parasite and not the IL-22^-/-^ deficiency *per se*. To test this, WT mice were infected with 5 or 10 *H*. *diminuta*, and while the latter did lead to increased spleen cell number and TH2 cytokine output, there were no differences in worm burden or intestinal IL-25 mRNA levels between the two infection paradigms at 8-dpi (S3 Fig). Thus, a higher antigenic load is not responsible for the increased IL-25 response but rather this is attributable to the absence of IL-22.

**Fig 4:**
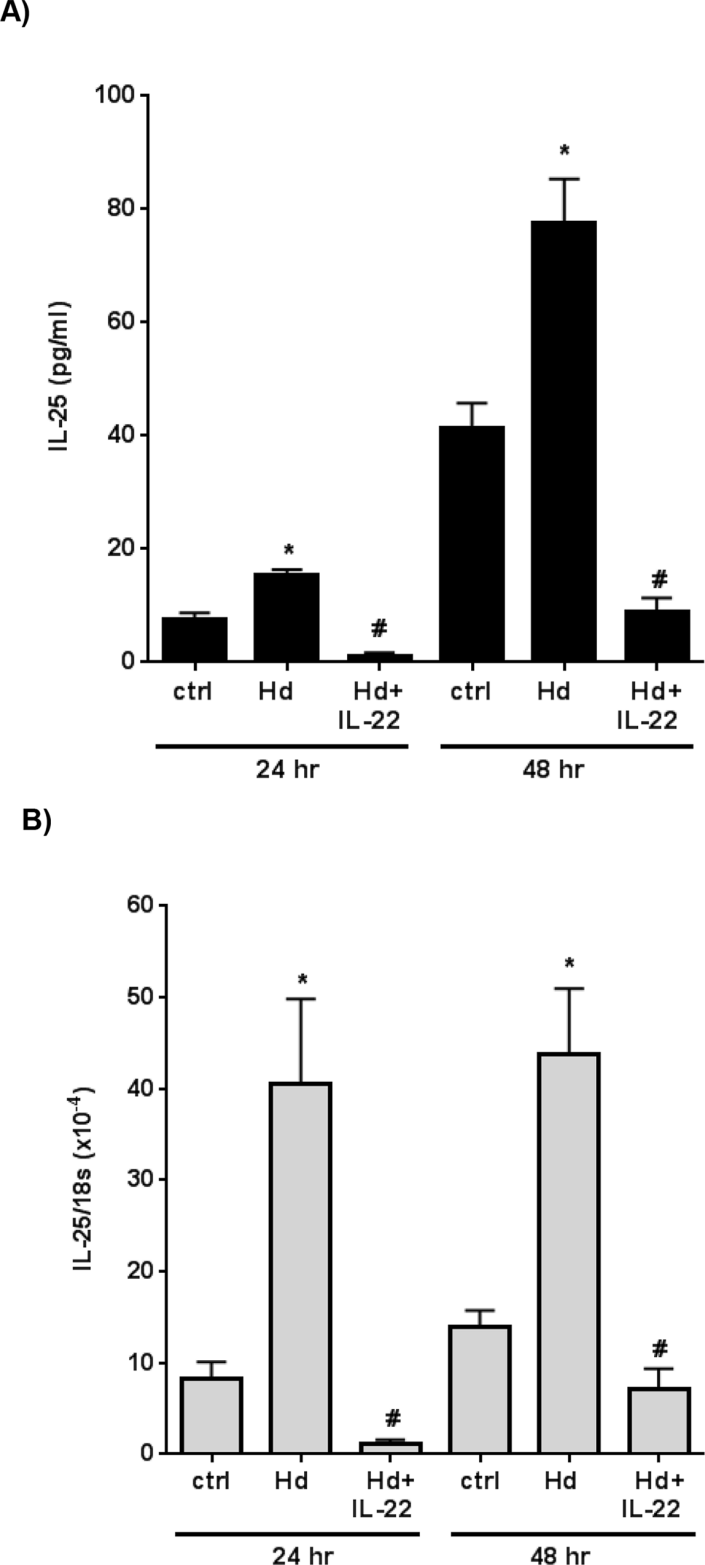
Interleukin 22 inhibits epithelial IL-25 production. Monolayers (~3 cm^2^) of the murine small intestinal IEC4 epithelial cell line were exposed to a single adult *H*. *diminuta* (*Hd*: scolex and 2 cm of strobila) ± recombinant IL-22 (5 ng/ml), and IL-25 measured in the culture medium by ELISA 24 and 48 hr later (Panel A). After collecting supernatants adherent cells were treated with Trizol, total mRNA obtained and IL-25 mRNA expression determined by qPCR (Panel B) (data are mean ± SEM; n = 6; * and #, p<0.05 compared to non-infected control (ctrl) cells and *Hd*-infected cells, respectively).

Focusing on IL-25, murine IEC4 epithelial cells were exposed to a single *H*. *diminuta* (scolex and ~2 cm of strobila) ± recombinant IL-22. Levels of IL-25 protein and mRNA expression were determined in supernatant and Trizol-treated cells, respectively. The epithelia spontaneously produced IL-25 that was significantly increased by *H*. *diminuta*, and in both cases IL-22 reduced IL-25 production (Fig 4A), correlating with mRNA levels (Fig 4B). To our knowledge this is the first time that IL-22 suppression of IL-25 production in the context of a parasitic helminth infection has been shown, underscoring the role of IL-22 in moulding the host response following infection. In addition, using the reductionist approach of culturing a single *H*. *diminuta* scolex with epithelial cell lines, we found epithelia from the non-permissive mouse host produced IL-25, IL-33 and TSLP (mRNA and protein) and that the rat (permissive host) IEC.6 cell line failed to show this alarmin response to the worm [36]. Of note, qPCR revealed a trend towards increased IL-33 and thymic stromal lymphopoietin (TSLP) mRNA expression in the jejunum of *H*. *diminuta* infected mice (S4 Fig); others have shown differential regulation of IL-25, IL-33 and TSLP following infection with helminth parasites [35].

In addition to its role as a TH2-polarizing cytokine, IL-25 inhibition of trinitrobenzene sulphonic acid (TNBS)-induced colitis in mice may involve alternatively activated macrophages (AAMs) [37], and markers of AAMs are increased in the gut of *H*. *diminuta*-infected mice [38]. Extrapolating from this, the increased IL-25 production from epithelia exposed to *H*. *diminuta* and the highly significant increase in IL-25 mRNA in the parasitised gut of IL-22^-/-^ could result in increased mobilization of immunoregulatory cells and suppression of concomitant disease in the infected mice.

**Fig 5:**
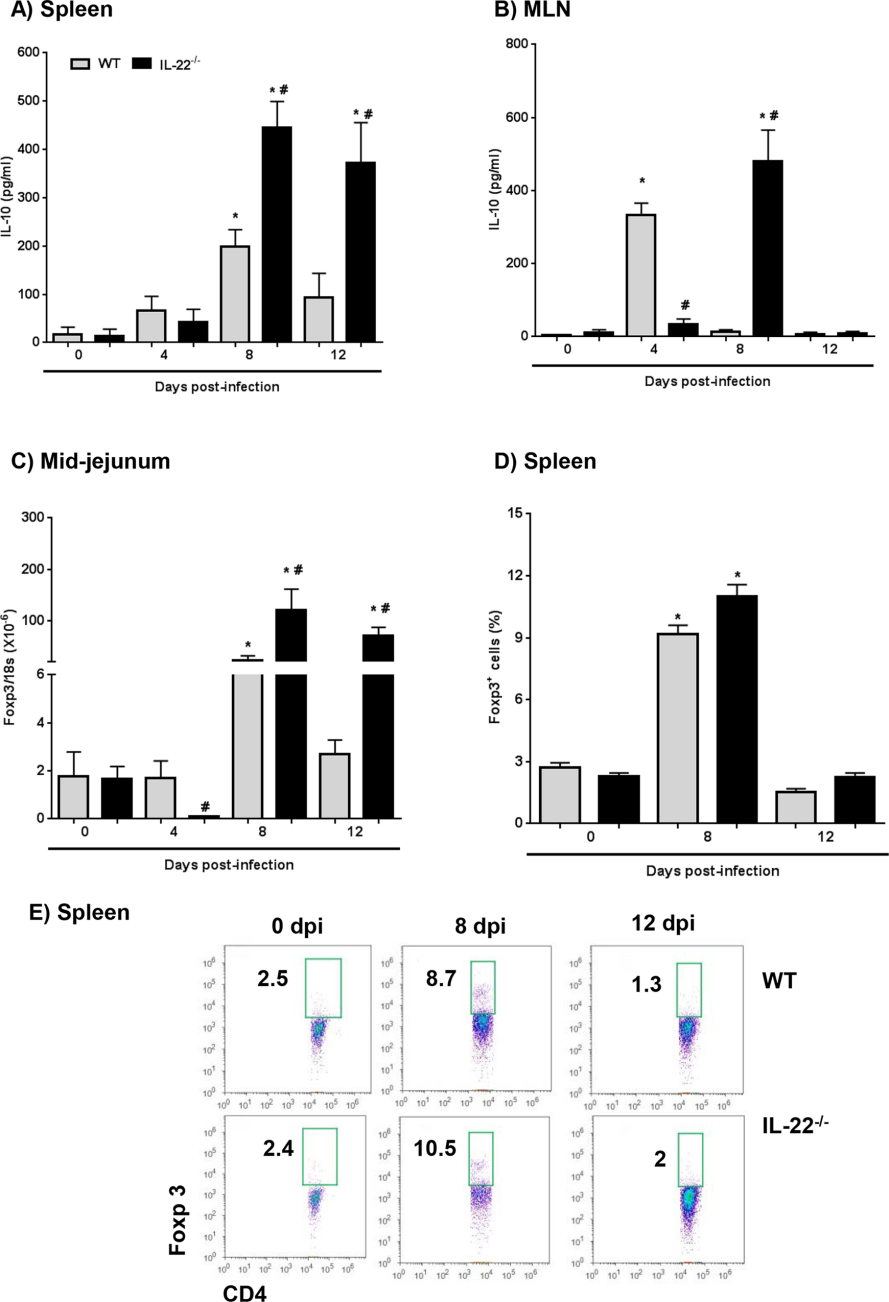
*H*. *diminuta*–infected IL-22^-/-^ display delayed and/or prolonged expression of immunoregulatory factors. Wild-type (WT) and IL-22^-/-^ mice were infected with 5 *H*. *diminuta* and at time-points thereafter splenocytes (A) and mesenteric lymph node cells (MLN) (B) were excised and stimulated with conA (5 μg/ml) for 48 hr and IL-10 measured. (C) Shows increases in Foxp3 mRNA in the mid-jejunum of WT and IL-22^-/-^ post-infection. (D) Flow cytometry revealed increased numbers of CD4^+^Foxp3^+^ splenocytes 8 days post-infection (dpi) in both WT and IL-22^-/-^ mice (E, representative dot plots) (data are mean ± SEM; n = 6–9; * and # p<0.05 compared to strain control and time-matched WT, respectively).

IL-10 synthesis follows infection with *H*. *diminuta* infection and is an important anti-inflammatory cytokine in mice and humans [23]. The increased levels of IL-10, Foxp3 and markers of AAMs (i.e. arginase-1 and Fizz1) mRNA found in the small intestine of *H*. *diminuta*-infected Balb/c mice [25], suggests expansion of innate and adaptive regulatory cells. These data were confirmed and extended here and, moreover, gut levels of IL-10 mRNA (Fig 2C) and stimulated IL-10 from splenocytes (Fig 5A) and MLN cells (Fig 5B) were significantly increased at 8- and 12-dpi in IL-22^-/-^ mice compared to WT mice. Macrophages can be an important source of IL-10 in response to helminth and microbial antigens [39, 40]. However, macrophages differentiated from the bone-marrow of IL-22^-/-^ mice had a normal capacity to produce IL-10 in response to *H*. *diminuta* antigen or LPS (S5 Fig); thus, we speculate that the increased IL-10 observed in MLN and splenocytes at the later time-points of infection in IL-22^-/-^ mice is from T cells, or potentially B cells [23, 41].

We have shown a variable increase in Foxp3 mRNA in the small intestine of *H*. *diminuta*-infected Balb/c mice [25]. Despite the likelihood of IL-22-Foxp3 cross-regulation [42] little is known of the putative interaction of these two factors following infection with helminth parasites. Increased Foxp3 mRNA was observed in the small intestine of IL-22^-/-^ *H*. *diminuta-*infected mice compared to WT animals (Fig 5C), supporting the notion that IL-22 serves as a brake on immunoregulatory cell mobilization; however, immunoblotting with extracts of small intestine failed to show a consistent increase in Foxp3^+^ cells, which may reflect sensitivity of this assay as compared to qPCR (S6 Fig). Moreover, while CD4^+^Foxp3^+^ splenocytes were increased following infection (8-dpi) there were no differences between WT and IL-22^-/-^ mice. (Fig 5D and 5E). The reason for the discrepancy between small intestine and spleen is unclear but it underscores the complexity of immunoregulation and the need to precisely define events in both time and space as they relate to the host response to infection. In addition, expression of Foxp3 does not unequivocally identify a cell with immunosuppressive capacity [43], and so IL-10 may be more important than Foxp3 in immunoregulation in this helminth-rodent model system [23].

Although the interaction of IL-25 and Foxp3 expression was not pursued, the association is noteworthy, given data showing lower numbers of Tregs in IL-25^-/-^ mice [44], increases in antigen-specific IL-22^+^ T cells concomitant with fewer Foxp3^+^ T cells in an individual with ulcerative colitis infected with *T*. *trichura* [22], and that NOD mice treated with IL-25 have increased numbers of Tregs [45]. Thus, one can speculate that the increase in IL-25 in IL-22^-/-^ mice could mediate the increase in Foxp3 and hence Tregs. The role of IL-22 in controlling the mobilization and activity of immunoregulatory cells is not well understood and in addition to considering Tregs, the putative impact of IL-22 on B cells should not be overlooked: for example, successful treatment of tuberculosis correlated with, but was not functionally linked, to increased IL-22 production and a reduced frequency of putative regulatory CD5^+^CD1d^+^ B cells [46].

### IL-22 participates in DNBS-induced colitis and restricts the anti-colitic effect associated with infection with *H*. *diminuta*

**Fig 6:**
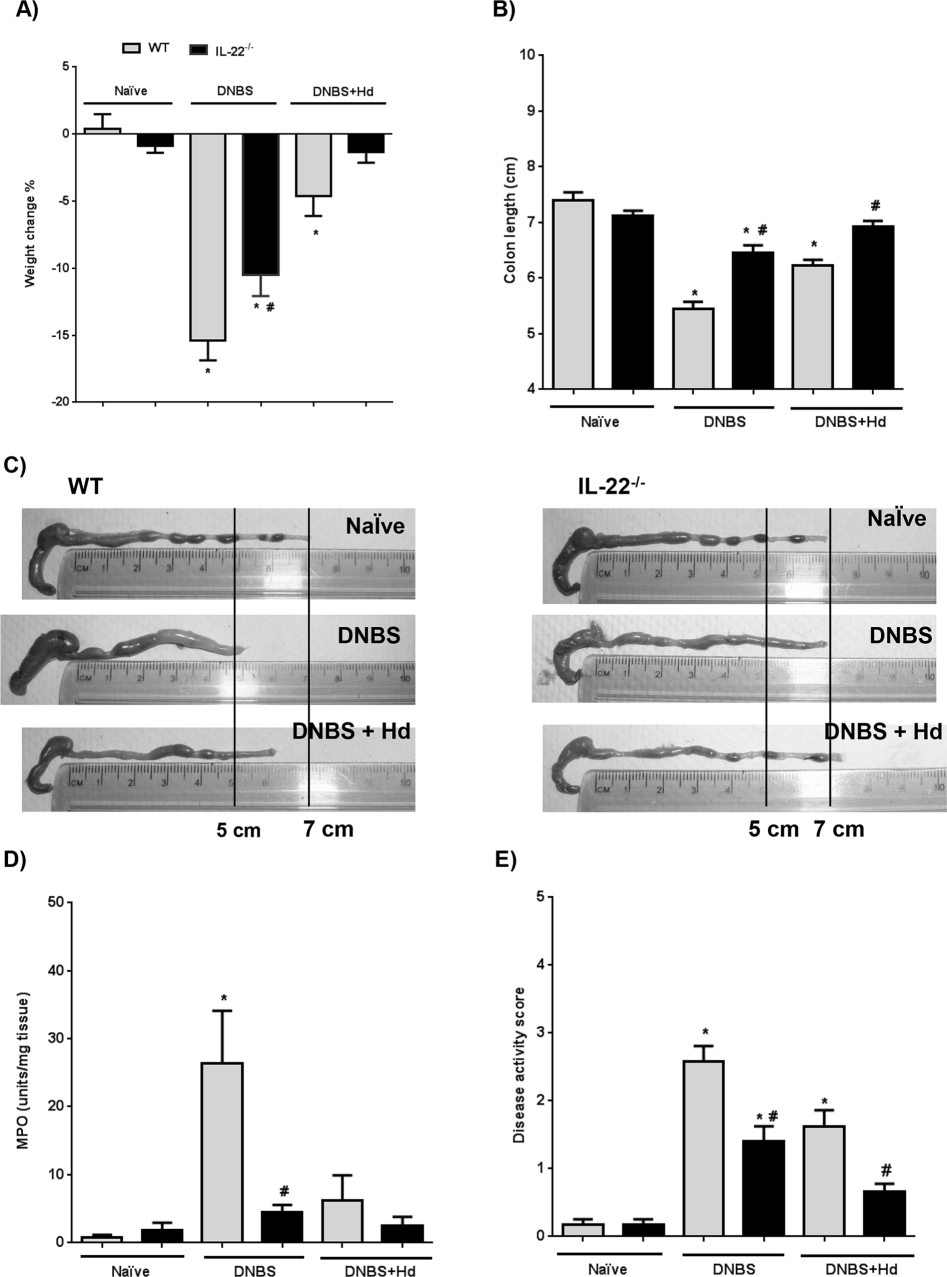
IL-22^-/-^ mice experience less DNBS-induced colitis and enhanced anti-colitic effects following infection with *H*. *diminuta*. Wild-type (WT, gray bars) and IL-22^-/-^ (black bars) mice were infected with 5 *H*. *diminuta* (*Hd*) cysticercoids and 8 days later were given DNBS (5 mg, ir). Mice were necropsied 72 hr post-DNBS and weight loss (A) and degree of colon shortening (B) were assessed for each group. In panel (C), representative colon images from experimental groups are shown. MPO activity in the distal colon was determined and presented as granulocyte marker (D), and overall disease activity scores (DAS) are shown in (E). Data are mean ± SEM; n = 9; * and # p<0.05 compared to strain control and time-matched WT, respectively.

Based on the changes observed in IL-22^-/-^ mice following infection with *H*. *diminuta*, we examined the impact of lack of IL-22 in (a) the outcome of DNBS-induced colitis and (b) the ability of infection with *H*. *diminuta* to reduce the severity of DNBS-induced colitis. IL-22^-/-^ mice consistently developed less severe DNBS-induced colitis compared to WT mice, in all indicies measured: weight loss, colon length and macroscopic appearance, MPO activity (indicative largely of neutrophil infiltration) and the cumulative disease activity score (DAS) (Fig 6). This effect was compounded following infection with *H*. *diminuta*: infected IL-22^-/-^ mice treated with DNBS showed minimal signs of disease and were often indistinguishable from control, non-DNBS-treated mice (Fig 6). These findings are in accordance with the reduced mobilization of IFNγ and neutrophils observed in *T*. *gondii*-infected IL-22^-/-^ compared to WT mice [47] ((DNBS-induced colitis is considered a TH1-dominated disease and hence the balance of TH1 and TH2 immunity is important in disease severity [48]).

**Fig 7:**
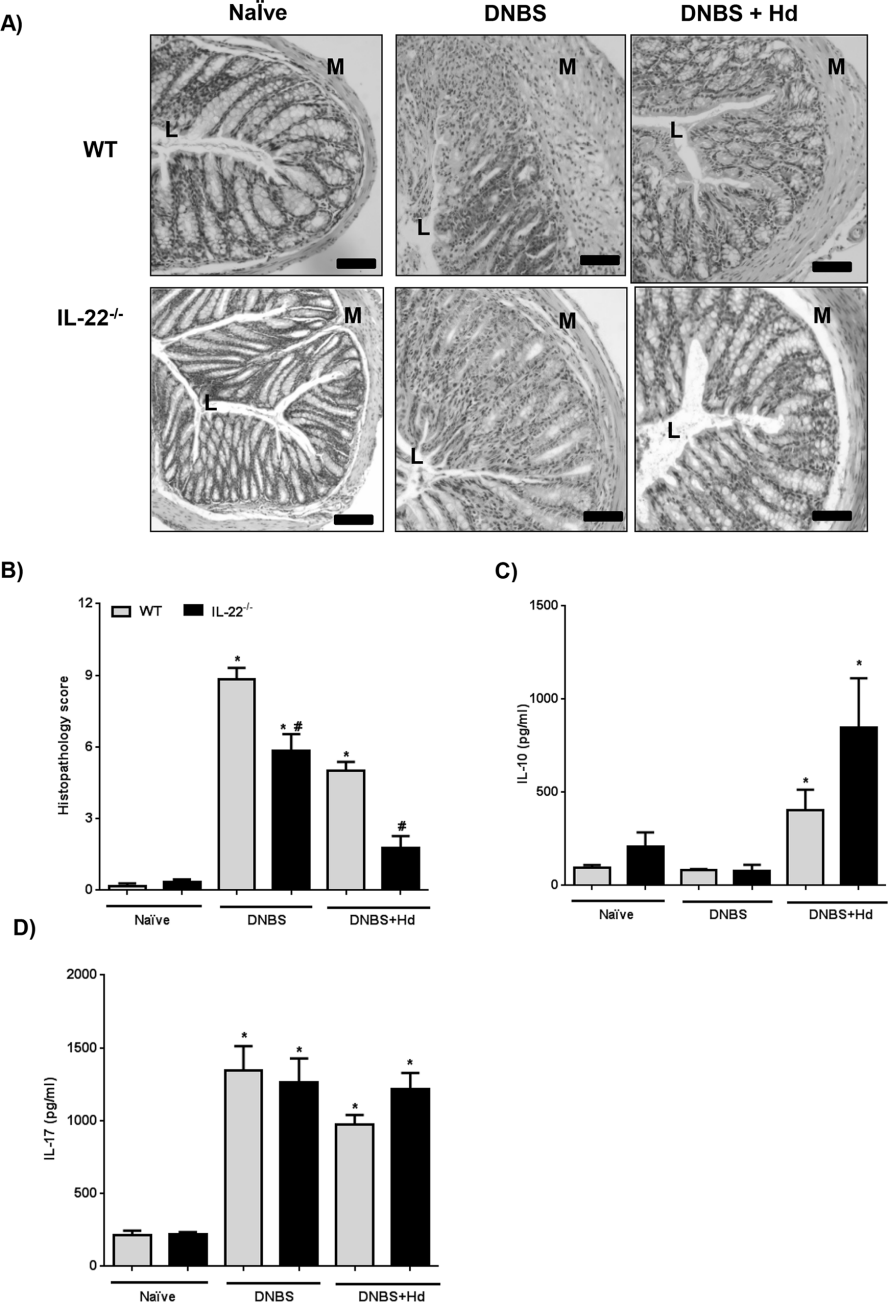
Mice lacking IL-22 show reduced DNBS-induced histopathology and increased splenic IL-10. Wild-type (WT, gray bars) and IL-22^-/-^ (black bars) mice were infected with 5 *H*. *diminuta* (*Hd*) and 8 days later were given DNBS (5 mg, ir). Mice were necropsied 72 hr post-DNBS and histopathology assessed on H&E stained sections (representative images shown in A) and scored on a 12-point scale in a blinded fashion (B). Spleen cells were stimulated *in vitro* for 48 hr with conA (5 μg/ml) and levels of IL-10 (C) and IL-17 (D) determined by ELISA (data are mean ± SEM; n = 6; * and # p<0.05 compared to strain control and time-matched WT, respectively; M, external muscle layers, L, lumen of colon, scale bar = 100 μm).

Corroborating these macroscopic measures of disease activity, histological analyses revealed that IL-22^-/-^ mice had less DNBS-induced histopathology compared to WT mice, and only very minor damage was observed in the colon of *H*. *diminuta*-infected IL-22^-/-^ mice (Fig 7A and 7B). Mitogen stimulation of splenocytes from WT or IL-22^-/-^ mice infected with *H*. *diminuta* revealed increased IL-10 production compared to uninfected mice, both naïve and DNBS-treated. Cells from infected DNBS-treated IL-22^-/-^ mice produced, on average, more IL-10 than WT mice, but this did not reach statistical significance (p = 0.2) (Fig 7C). In contrast, splenic production of IL-17, while increased by DNBS, was not significantly different between WT and IL-22^-/-^ mice ± infection with *H*. *diminuta* (Fig 7D). Juxtaposing these data with those from *H*. *diminuta*-infected naïve IL-22^-/-^ (Figs 2–5), it is likely that the increase in IL-10, IL-25 and putative regulatory T cells (i.e. increased jejunal Foxp3 mRNA) enhances the anti-colitic effect of infection with *H*. *diminuta* in mice lacking IL-22.

It has been reported that IL-22 protects female Balb/c mice from TNBS (3 mg, 5 days)-induced colitis [49] (yet others found no increase in IL-22 mRNA in TNBS-treated animals [50]). Given the structural similarity of DNBS and TNBS how can these disparate roles of IL-22 be reconciled? Differences in the sex of mice, the duration of the disease and the natural microbiota of the mice could, at least in part, underlie the opposing findings of the two studies. Also, the protective effect of IL-22 in TNBS-colitis was based on administration of a neutralizing antibody and not genetic knockout of the IL-22 gene, raising the possibility of non-IL-22 effects of the antibody. Again, the point arises that the beneficial versus detrimental impact of manipulating IL-22 as a therapy will be contextual.

IL-22^-/-^ mice have increased susceptibility to dextran sodium sulfate (DSS)-induced colitis [4] and hence the findings in the DNBS model were somewhat surprising. We confirmed that the IL-22^-/-^ mice used here had heightened responsiveness to DSS (S7 Fig). The increased severity of DSS-induced colitis in IL-22^-/-^ mice has been linked to a pro-colitiogenic microbiota [4]. To address this, a published protocol [51] was used to blend the microbiotas between WT and IL-22^-/-^ mice prior to DNBS treatment. The severity of colitis in IL-22^-/-^ mice with their natural microbiota and those who acquired microbiota from WT mice was not different, and both had significantly less disease than WT mice (S8 Fig). In contrast, all of the WT mice who acquired microbiota from IL-22^-/-^ mice presented with severe DNBS-induced colitis, with a marked increase in the size of the cecum: these mice were the sickest of all the experimental groups (S8 Fig). Thus, IL-22^-/-^ mice may harbour a microbial pathobiont that is not important to DNBS-induced colitis in these mice but exaggerates disease in WT mice, somewhat analysis to the transmissibility of susceptibility to DSS by the microbiota from IL-22^-/-^ mice [4]. Assessing the possibility that IL-22^-/-^ could be deficient in anti-microbial peptides, qPCR revealed that this was not the case. In line with findings reported in intestinal bacterial infection increases in mRNA for β-defensin 1, 2 and 3 was similar in WT and knock-out mice following infection with *H*. *diminuta* (S9 Fig). Interestingly, unlike infection with *C*. *rodentium* that increased RegIIIβ and RegIIIγ in a IL-22-dependent manner [14], infection with *H*. *diminuta* evoked only a transient increase in RegIIIβ but not RegIIIγ mRNA (S9 Fig). Thus, the contribution of IL-22 to DNBS-induced colitis is not likely due to different microbiota rather it is a consequence of altered immunoregulation in the absence of IL-22.

The fact that IL-22^-/-^ mice experience less DNBS-induced and greater DSS-induced colitis highlights important differences in disease pathogenesis. Up-regulation of IL-22 mRNA has been found in DSS- but not in TNBS-induced colitis [50]. In the gut, T cells, γδ T cells and ILC3s are major sources of IL-22 [52]. More recently neutrophils have been cited as a source of IL-22 [53]. However, the extent to which each cell is activated in colitis and by which stimuli (i.e. cytokines vs. pattern-associated microbial patterns) is not fully understood. Consequently additional efforts are required to unravel the role of IL-22 in a variety of model systems and in the context of varying microbiotas if extrinsic manipulation of IL-22 levels is to be considered a treatment for enteric disease.

The situation is complicated further by the recent demonstration that IL-25^-/-^ mice are protected from DSS-induced colitis [54] (anti-IL-25 neutralizing antibodies can inhibit oxazolone-induced colitis [55]). Thus, application of anti-IL-22 or anti-IL-25 antibodies to manipulate human disease would need to proceed with caution and be preceded by precise work-up of the immunological basis of the disease in the patient to be treated.

### *In vivo* immunoneutralization of IL-25 in IL-22^-/-^ mice reverses the reduced susceptibility to DNBS-induced colitis

The role of IL-25 has been assessed in TH2-mediated airways diseases as an early TH2-promoting factor [56–58]. In the context of TH1-mediated pathologies, IL-25 has been shown to suppress IL-17 and IFNγ production in infectious [26] and autoimmune diseases (e.g experimental autoimmune encephalitis (EAE) [59] and diabetes [45]). Interleukin-25 has been found to inhibit the release of IL-1β, IL-12(p40) and TNFα from LPS-activated human CD14^+^ monocytes [60] which could in part explain its’ suppression of TH1-driven immunopathologies.

**Fig 8:**
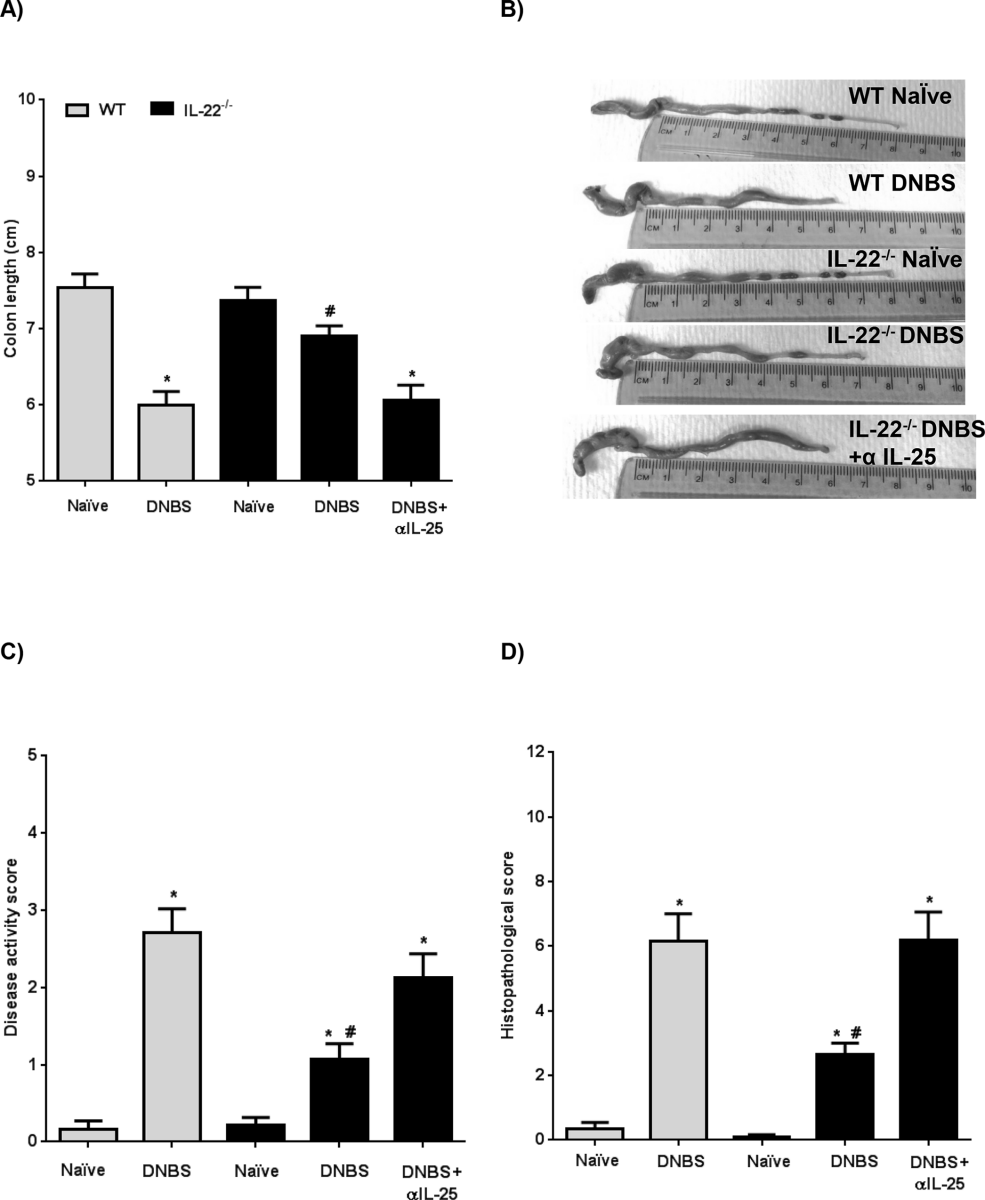
Resistance to DNBS colitis in IL-22^-/-^ mice requires IL-25. Wild-type (WT; grey bars) and IL-22^-/-^ (black bars) mice received 5 mg of DNBS intrarectally to induce colitis, with some IL-22^-/-^ also receiving anti-IL-25 blocking antibody (100 μg/mouse, ip., given 10 minutes before DNBS delivery). Severity of colitis was assessed 72 hr post-DNBS by (A, B) measurement of colon length, (C) disease activity score and (D) histological damage score (data are mean ± SEM; n = 6–8; * p<0.05 compared to appropriate strain-matched control mice and # p<0.05 compared to animals given anti-IL-25 blocking antibody).

Having found increased IL-25 expression in *H*. *diminuta*-infected IL-22^-/-^ mice and that these mice were highly resistant to DNBS-induced colitis, a causal relationship between these two observations was tested via administration of IL-25 neutralizing antibodies [61]. First, the role of IL-25 during DNBS-colitis in IL-22^-/-^ mice in the absence of *H*. *diminut*a infection was addressed. Consistent with the previous data, IL-22^-/-^ mice displayed less severe DNBS-induced colitis compared to WT mice (Fig 8). However, IL-22^-/-^ mice treated with DNBS and anti-IL-25 blocking antibodies had a severity of colitis that was macroscopically (Fig 8A–8C) and microscopically (Fig 8D) indistinguishable from WT mice that received DNBS only. Thus, in the absence of infection with *H*. *diminuta* (an IL-25 trigger), IL-22 represses IL-25 during inflammatory responses induced by DNBS and when IL-25 is blocked the resistant phenotype observed in IL-22^-/-^ mice is negated.

**Fig 9:**
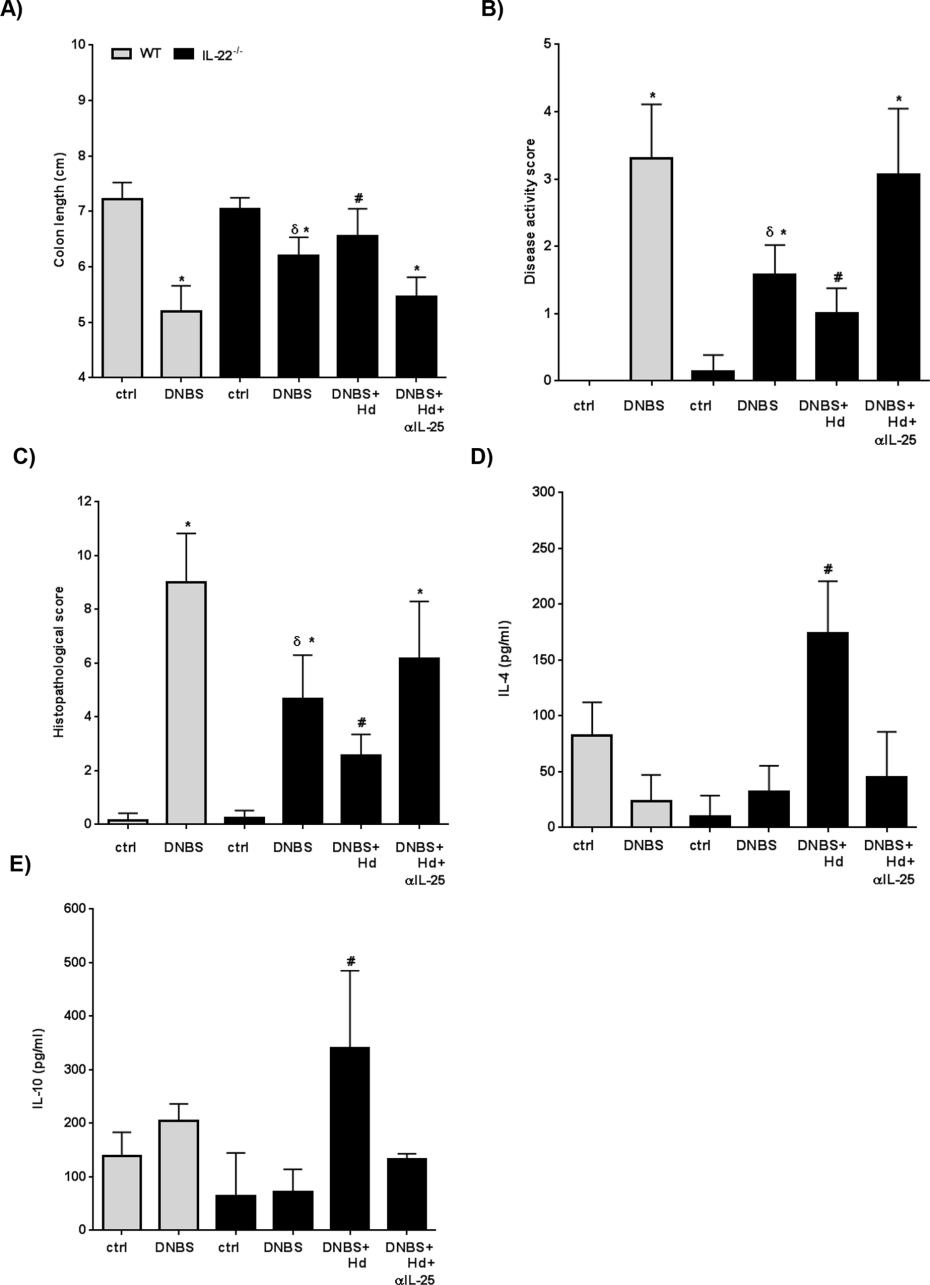
Interleukin-25 is a major player in the anti-colitic effect in *H*. *diminuta* infected IL-22^-/-^ mice. Interleukin-22^-/-^ mice were orally infected with 5 cysticercoids of *H*. *diminuta* and 8 days later received a single ip. injection of anti-IL-25 antibody (100 μg) concomitantly with delivery of DNBS (5 mg, ir.). Necropsy was performed 72 hr later and severity of colitis determined by (A) colon length, (B) macroscopic disease activity score, (C) histopathology scores, and conA stimulated splenocytes production of (D) IL-4 and (E) IL-10 (data are mean ± SEM; n = 6–8 from 2 independent experiments, except panel E where n = 3–5; * and δ p<0.05 compared to appropriate strain-matched control mice and wild-type (WT) DNBS, respectively) and # p<0.05 compared to animals infected and given anti-IL-25 blocking antibody.

*In vivo* immunoneutralizing of IL-25 in DNBS+*H*. *diminuta*-infected IL-22^-/-^ mice resulted in a severity of colitis that was similar to DNBS-only treated mice, indicating a requirement for IL-25 in the anti-colitic effect evoked following infection with this helminth (Fig 9). These findings complement other studies in which IL-25 has been shown to down-regulate inflammatory gut disease: for example colitis induced in mice by bacterial peptidoglycan, TNBS, oxazolone or DSS [37, 62, 63]. Going forward it will be intriguing to test helminth therapy with/without IL-22 in chronic models of colitis and those driven by adaptive immunity such as the naïve T cell transfer model [4].

Assessment of the role of IL-22 in immunity and inflammation reveals that the impact of this cytokine is highly contextual, with convincing evidence in favour of anti- and pro-inflammatory roles [4, 5, 7, 64]. While many of the functions of IL-22 in the gut promote protective anti-microbial responses, a pathogenic role for IL-22 has been described following infection with *T*. *gondii* [19] and *Helicobacter pylori* [65]. Less is known of the role of IL-22 in the host response to infection with helminth parasites. Increases in local IL-22 or IL-22^+^ cells have been described in response to gastrointestinal helminths [21, 22], yet the function of IL-22 was inferred not tested. The notable exception being the demonstration of impaired expulsion of nematodes in IL-22^-/-^ mice that aligned with reduced goblet cell hyperplasia [20]. The role of IL-22, if any, in regulating the response to cestode parasites has not hitherto been examined. Production of IL-22 can be evoked by IL-9, IL-23 and microbial stimuli [3] and while IL-25 suppression of IL-22 has been shown [66], less is known of the reciprocal interaction. We have found that increases in IL-25 mRNA in the parasitized intestine and IL-25 synthesis by enteric epithelia exposed to *H*. *diminuta* are suppressed by IL-22. This is, to our knowledge, the first time IL-22 has been directly implicated in the control of helminth-evoked IL-25, and complements earlier work showing that IL-22 inhibited IL-25 production by cytokine-treated murine airways epithelia [67].

**Fig 10:**
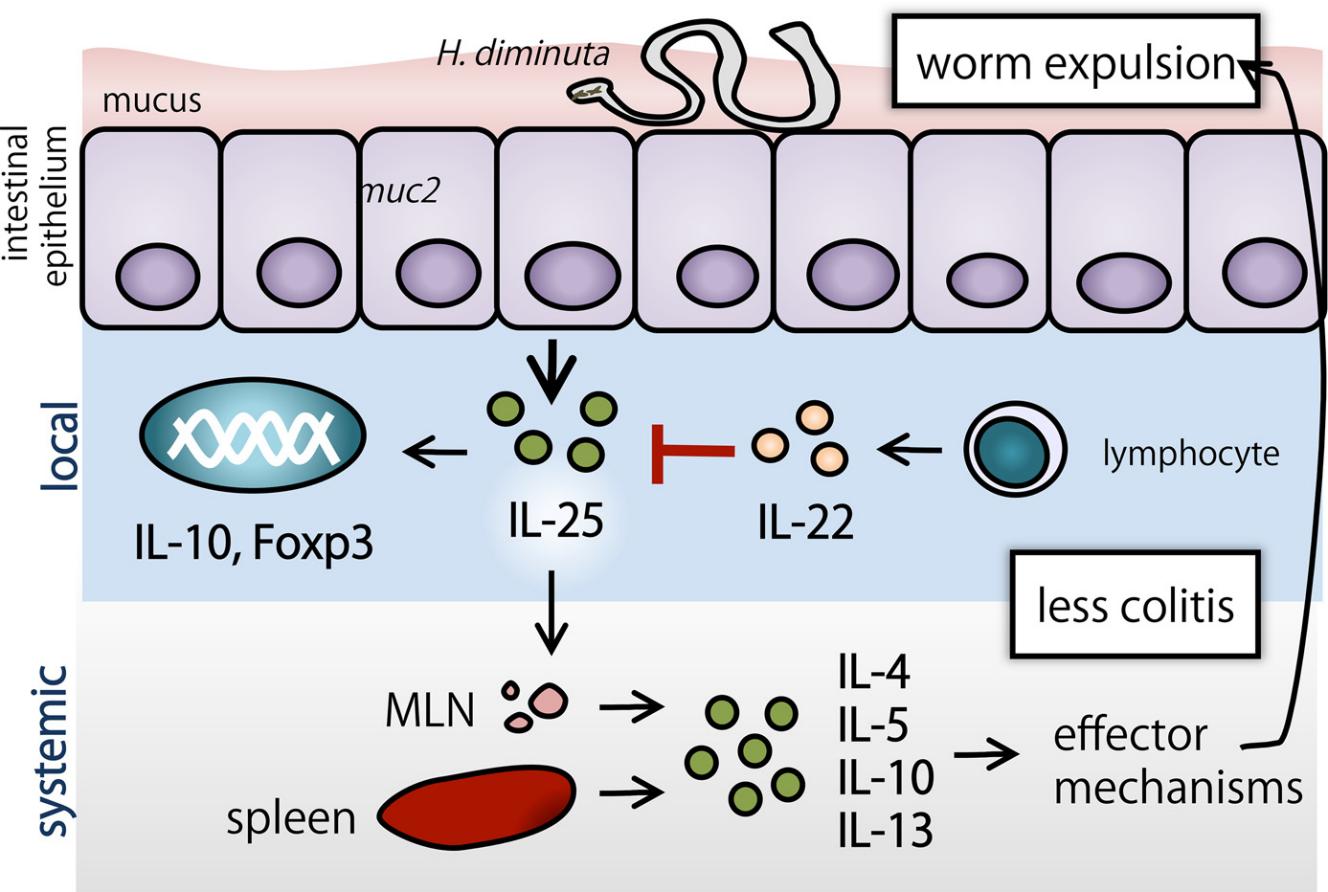
Proposed model of immune response upon *H*. *diminuta* infection in mice lacking IL-22. The presence of *H*. *diminuta* provokes an increase in TH2 and regulatory populations both locally (small intestine) and systemically (MLN and spleen), which ultimately leads to worm expulsion in a non-permissive host (mouse). This regulatory network is responsible for blocking DNBS-colitis. Herein, we identified a heightened regulatory circuit in *H*. *diminuta* infected mice lacking IL-22.

Using the *H*. *diminuta*-mouse model system, data have been obtained that support the following conclusions: (1) absence of IL-22 reduces the early TH2 response to infection with helminth parasites, suggesting an important initial role for innate immunity against metazoan parasites; (2) IL-22 is an endogenous brake on helminth-provoked TH2 immunity, and in its’ absence there is heightened/prolonged local (i.e. gut) and systemic TH2 and immunoregulatory events (e.g. IL-10), likely driven in large part by the increase in IL-25; and, (3) by limiting the synthesis of IL-25, IL-22 participates in the pathogenesis of DNBS-induced colitis and restricts the *H*. *diminuta*-suppression of colitis (Fig 10). Helminth therapy has been presented as a novel approach to auto-inflammatory disease [68] and we speculate that precise knowledge of the immunological basis of the disease would be important in selecting patients for helminth therapy.

## Methods

### Mice, parasites and infection

Interleukin-22 deficient mice (IL-22^-/-^: C57BL/6 background) were bred at the University of Calgary (pairs kindly provided by Dr. M. Kelly (Univ. of Calgary)). Mice were housed in a 12:12 hr light:dark cycle with free access to food and water and 8–9 weeks old male IL-22^-/-^ and age-matched C57BL/6 control mice (Charles River, QB, Canada) were used throughout this study. As defined in the experiments, mice received 5 infective *H*. *diminuta* cysticercoids in 100 μl of sterile 0.9% NaCl by oral gavage and 8 days later colitis was induced [23]. In one experiment doses of 5 and 10 *H*. *diminuta* cysticercoids were compared.

### Ethics statement

All experiments were conducted following the regulations specified by the Canadian Guidelines for Animal Welfare and were approved by the University of Calgary Health Science Animal Care Committee (HSCCC) with the protocol number AC13-0015.

### Worm recovery

At time-points post-infection, the small intestine was excised and flushed with 2 ml of 4°C PBS. The intestine was opened longitudinally and examined along with the flushed contents for *H*. *diminuta*.

### Induction of experimental colitis and evaluation

Colitis was induced by intrarectal (ir.) installition of 5 mg/mouse of DNBS (MP Biomedicals Ohio, USA) in 100 μl of 50% ethanol 3 cm into the colon. Weight was recorded daily for 3 days, the mice humanely necropsied and a macroscopic disease activity score on a 5 point scale based on weight loss, colon shortening, stool consistency and general appearance determined as previously [23]. A portion of mid-colon was excised, formalin fixed, paraffin embedded and 5 μm sections were collected on coded slided, stained with hematoxylin and eosin and a histopathology score determined on a 12-point scale [23]. The most distal 1 cm of colon was snap frozen in liquid nitrogen for myeloperoxidase (MPO) determination as measure of granulocyte, mainly neutrophil, infiltrate. MPO activity was determined by a kinetic assay in which H_2_O_2_ catabolism is measured, and 1 unit of MPO activity is the amount of enzyme required to degrade 1 μM of H_2_O_2_/min [23].

In other experiments, a 5 day exposure to 2.5% wt./vol. DSS (MW: 30,000–50,000; MP, Biomedicals, OH, USA) was used to induce colitis. Mice were transferred to regular tap water on day 5, and 3 days later were assessed for disease severity as described above.

### Goblet cell and mast cell staining

Formalin-fixed, paraffin-embedded mouse mid-small intestine was sectioned (5 μm), sections collected on coded slides and stained with periodic-acid Schiff’s stain to identify goblet cells [31]. Cells were counted on a per villus-crypt unit (VCU) basis, as defined by an intact, rounded villus tip and an even layer or enterocytes indicating lack of obligue sectioning.

To identify mast cells, sections were deparaffinized followed by epitope retrieval with 10 mM sodium citrate buffer pH 6.0. After washing sections were incubated in PE anti-mouse CD117 (c-Kit) antibody (BioLegend, CA, USA) (1:500) in blocking solution at 4°C overnight. Subsequently sections were washed in PBS, incubated in DAPI (0.1 μg/mL, 10 min. at room temperatura) and after a final PBS wash, slides were mounted using ProLong Gold (Cell Signaling Technology) and examined with a Nikon 80i microscope and DXM1200C camera. Images were captured using NIS-Elements software (Nikon), and representative images were processed in Adobe Photoshop (Version 8.0).

### Measurement of systemic immune response

At indicated times the spleen and mesenteric lymph nodes (MLN) were asceptically removed from WT and IL-22^-/-^ *H*. *diminuta*-infected mice, cell suspensions generated and red blood cells lysed in ammonium chloride buffer [23]. Cells were adjusted to 3x10^6^ /ml in RPMI 1640 medium supplemented with 10% FBS, 0.1 mM (Gibco, USA). Cells were activated by treatment with concanavalin A (5 μg/ml) and 48 hr later supernatants were collected and stored (-80°C) for cytokine measurements by ELISA.

### ELISA sandwich

Interleukin (IL)-4, IL-5, IL-10, IL-13, IL-17, IL-25 and IFNγ were measured by ELISA using paired antibodies and following the manufactures’ instructions (R&D Systems Inc., Minneapolis, USA). All samples were measured in duplicate and assays had dectection limits that ranged from 2–9 pg/ml.

### Flow cytometry

Spleens were aseptically excised and cell suspensions generated as above. Thereafter, 1x10^6^ splenocytes were incubated with TrueStainX (anti-CD16/32) for 10 min at 4°C and then stained. Cells were stained for 30 min with conjugated APC-CD4 (Biolegend, San Diego, CA USA). After incubation with APC-CD4 antibody cells were washed in flow buffer (PBS, 1%FBS and 0.1% NaN_3_) and intracellular staining for Foxp3 was performed following manufacturer’s protocol. Briefly, after surface staining cells were washed with flow cytometry buffer, then fixed and permeabilized with Foxp3 Fix/Perm and Foxp3 Perm buffers respectively. A final incubation with Foxp3-AlexaFluor 488 (Biolegend, San Diego, CA) was conducted for 30 min at room temperature in the dark. Data were acquired in a Attune cytometer and analyzed with Attune V.6.1 software (R&D systems).

### qPCR in intestine

Small intestine was excised from non-infected and *H*. *diminuta*-infected WT and IL-22^-/-^ mice, flushed with 4°C PBS, and the 3 cm portion of mid-intestine was cut in three pieces, placed in 1ml of TRizol Reagent (Invitrogen, California, USA) and homogenized for 60 seconds (Polytron MR2100, Kinematica AG, Switzerland). The RNA was extracted with chloroform/ethanol as previously [25] and 1 μg of RNA was used as the template for cDNA generation with the iScript DNA synthesis kit (Bio-Rad, USA). Conditions for the PCR were denaturation 95°C for 2 min, 40 amplifying cycles of 95°C 15 sec, 55°C 15 sec, 68°C 20 sec and final temperature 4°C; primer sequences are presented in S10 Fig.

### Immunoblotting for Foxp3

At indicated times after *H*. *diminuta* infection ~1cm of jejunum was excised and homogenized in RIPA buffer (50 mM Tris-HCl, 150 mM NaCl, 1% NP-40 0.5% sodium deoxycholate and 0.1% SDS) supplemented with protease inhibitor cocktail (Promega, Madison Wisconsin USA). Protein concentration was determined by the Bradford assay (Bio-Rad Laboratories Mississauga ON, Canada). Samples were normalized to 10 μg protein/μl and run by SDS-page (4% stacking, 8% separating) and transferred to a nitrocellulose membrane. Membranes were blocked for 1 hr at room temperature in 5% skim milk in 0.1% TBS-tween buffer and then incubated overnight with purified anti-Foxp3, 3 μg/ml (Biolegend, California, USA). After washing, membranes were incubated with appropriate secondary antibody for 1 hr at room temperature and developed by exposing to western lightning plus enhanced chemiluminiscence solution (PerkinElmer, Woodbridge ON, Canada) for 1 min and using an automatic film developer.

### Exposure of IEC4 cells to live *H*. *diminuta*

The mouse small intestinal epithelial cell line, IEC4, was maintained by serial passage in DMEM medium supplemented with HEPES (1%), L-glutamine (10%), Pen/Strep (1%) and FBS (5%) (all from Gibco, USA). One-million IEC4 cells were seeded in 6-well plates and cultured for 48 hr. Scolices and 2 cm of strobila of *H*. *diminuta* retrieved from the small intestine of rats or IL-4 receptor-α^-/-^ mice (fail to expel *H*. *diminuta*) were exposed to a cocktail of antibiotics (Gentamicin solution, Sigma, St. Louis, Mo, USA) for 2 hr. A single worm was added to IEC4 monolayers ± recombinant IL-22 (5 ng/ml; Biolegend, CA, USA), and supernatants collected for measurement of IL-25 and then total RNA extracted.

### *In vivo* IL-25 neutralization

To determine the role of IL-25, IL-22^-/-^ were treated with a single ip. injection of 100 μg of an anti-IL-25 blocking antibody (clone 35B, Biolegend, CA, USA) ~10 min prior to DNBS ir. delivery and the severity of colon inflammation was assessed 72 hr later (as above [23]).

### Transfer of colonic bacteria

Following a protocol to transfer colonic microbiota between mice [51,69], WT and IL-22^-/-^ mice were transferred to cages with fresh bedding and 24 hr later mice were swapped into the opposing strains cage without a bedding change for 24 hr (coprophagy allows blending of the microbiota between the two strains). This cycle of swapping mice between cages was continued for 2 weeks. On day one of the procedure all mice were treated with kanamycin (40 mg/kg), gentamicin (3.5 mg/kg), colistin (4.2 mg/kg), metronidazole (21.5 mg/kg) for 3 days in their drinking water followed by an ip. injection of vancomycin (4.5 mg/kg). On day 15 mice were anesthetized and given DNBS (5 mg/mouse) intrarectally and colitis severity was assessed 72 hr later.

### Bone marrow derived macrophage development and *in vitro* stimulation

Bone marrow was flushed from the long bones of the legs of WT and IL-22^-/-^ mice via a sterile 27 gauge needle, the red blood cells lysed and the cells were incubated in RPMI 1640 medium (Gibco, USA) supplemented with 20% FBS, HEPES, Glutamax and antibiotic (Penicillin-streptomycin Sigma, St. Louis, Mo, USA) for 7 days in presence of 20 ng/ml murine M-CSF. On days 2 and 4 cells were treated with fresh medium containing macrophage-colony stimulating factor (M-CSF). At day 7, mature macrophages were harvested and seeded at 2.5x10^5^ in 24-well plates in above-mentioned medium and incubated with PBS-soluble crude *H*. *diminuta* antigen (*Hd*Ag: 100 μg/ml [70]) for 24 hr. As additional control, macrophages were also stimulated with LPS (10–1000 ng/ml (Sigma, St. Louis, MO, USA)). Supernatants were collected and assayed for TNFα by ELISA.

### Statistical analysis

Data are presented as mean ± the standard error of the mean (SEM) and statistical differences were determined by one-way ANOVA followed by post-hoc analysis with Student’s *t* test or Kneuman’s Keuls test and p<0.05 accepted as a statistically significant difference (Graph Pad prism V5 software, La Jolla, CA, USA).

## Supporting Information

S1 Fig Levels of TH1 cytokine IFN γ decrease during *H*. *diminuta* infection and remain unaltered in absence of IL-22. At 4, 8 and 12 days post infection spleen from infected experimental groups were collected, RBCs depleted and cell suspensions generated. Cell suspensions were incubated for 48 hr in presence of conA (5 μg/ml) and supernatants collected. Levels of IFN γ were determined by ELISA as described in methods. Data shown are mean ± SEM from independent experiments where * p<0.05 as compared to strain-matched control and # p<0.05 compared to WT time-matched group (n = 7).
(TIF)

S2 Fig Mast cells numbers are not significantly different in *H*. *diminuta*-infected wild-type (WT) or IL-22^-/-^ mice. Mice were necropsied at the days post-infection (dpi) indicated, ~1cm of mid-jejunum was collected, fixed, paraffin embedded and immuno-staining performed with anti-cKit antibody (mast cell marker), as per the manufacturer’s instructions, and DAPI staining used to identify nuclei. Random fields of view were chosen based on DAPI staining and observed in a blinded fashion (images are representative of n = 3–4 mice; original mag. = x200).
(TIF)

S3 Fig High parasite burden results in increased cellular response but comparable IL-25 mRNA expression in small intestine. Wild-type mice received 5 or 10 cysticercoids of *H*. *diminuta* and on necropsy 8 days later (A) there was no difference in worm expulsion, while (B-D) the number of splenocytes and concanavalin-induced IL-4 and IL-10 production was significantly increased. (E) However, analysis of mid-jejunum segments extracted in Trizol by qPCR revealed no differences in IL-25 mRNA expression. Lines represent mean ± SEM; n = 5; * p<0.05 as compared to animals infected with 5 cysticercoids.
(TIF)

S4 Fig Tissue-derived cytokines IL-33 and TSLP are not modified due to IL-22 absence. Total mRNA was extracted from small intestine on indicated times after *H*. *diminuta* infection from both WT and IL-22^-/-^ mice and (A) IL-33 and (B) TSLP transcripts were measured and normalized against the housekeeping gene 18s. Data shown are mean ± SEM from 2 independent experiments (n- = 6).
(TIF)

S5 Fig Macrophages from IL-22^-/-^ mice do not over-produce IL-10 in response to *H*. *diminuta* antigens. Bone marrow precursors from WT and IL-22^-/-^ mice were differentiated into macrophages for 7 days as described in methods. Upon additional 24 hr of stimulation, supernatans were collected and levels of IL-10 in response to *H*. *diminuta* crude antigens (A) and LPS (B) were determined by ELISA. Data shown are from 2 independent experiments with similar results (n = 6).
(TIF)

S6 Fig Protein levels of Foxp3^+^ showed no increase in IL-22-/- mice compared to WT counterparts. At indicated times post-infection small intestine tissue from both WT and IL-22^-/-^ mice was homogenized in RIPA buffer and total protein extraction was conducted as indicated in methods and Foxp3 protein levels were determined. Beta-actin was used as loading control. Image is representative of 2 experiments with similar results.
(TIF)

S7 Fig IL-22^-/-^ mice are more susceptible to DSS-induced colitis than WT mice. Wild-type (WT) and IL-22^-/-^ mice were exposed to 2.5% (wt./vol.) dextran sodium sulfate (DSS) for 5 days followed by 3 days of normal drinking water and on necropsy IL-22^-/-^ mice displayed increased disease severity as assessed by (A) weight loss, (B) colon length, and (C) disease activity scores (DAS) (data are mean ± SEM; n = 5; * and #, p<0.05 compared to appropriate strain control (ctrl) and WT DSS mice, respectively).
(TIF)

S8 Fig Resistance to DNBS-induced colitis observed in IL-22^-/-^ mice is not due to their microbiota. The microbiotas were blended (Mix. Mic.) between wild-type (WT; gray bars) and IL-22^-/-^ mice (black bars) by cross-cage exchange and exploiting the coprophagic behavior of mice, followed by DNBS (5 mg, ir, 72 hr) treatment. The mixed or blended microbiota in IL-22^-/-^ mice did not affect their susceptibility to DNBS, with both groups having less severe colitis than WT mice assessed by colon shortening (A) and overall macroscopic score (B). Representative colon images in (C) show a reduced severity in IL-22^-/-^ mice regardless of having acquired microbiota from WT mice. In contrast, WT mice receiving microbiota from IL-22^-/-^ mice had the most severe colitis. Also, analysis of blind-scored H&E colon sections (D), confirmed less histopathological damage in absence of IL-22. Data are mean ± SEM; n = 5; * and #, p<0.05 compared to the appropriate strain matched control naïve mice and WT DNBS mice, respectively; arrow indicates enlarged caecum.
(TIF)

S9 Fig IL-22^-/-^ mice did not show impaired defensin and RegIII peptides expression during *H*. *diminuta* infection. Mid-jejunum tissue was homogenized in Trizol at the indicated time points and mRNA extracted as described in methods. Gene expression of Defensins 1–3 (A) and Reg III beta and gamma peptides was determined by using the specific primers quoted in S10 Fig Data are mean ± SEM from 2 independent experiments (n = 6), p< 0.0.5 as compared to expression found in wild-type (WT) animals.
(TIF)

S10 Fig Primer sequences used to determine gene expression of highly relevant players of intestinal immunity. Sequences were syntethized in Univ. of Calgary DNA core facilities or when indicated (i.e. β defensin 2 and β defensin 3) sequences were obtained as ready-to-use primer assay from Qiagen. Muc; mucin, Reg; Regeneration islet-derived protein.
(TIF)
