# Supplementary figures and images for: IL-22 Restrains Tapeworm-Mediated Protection against Experimental Colitis via Regulation of IL-25 Expression
Source: PLoS Pathog. 2016 Apr 7;12(4):e1005481. doi: 10.1371/journal.ppat.1005481 (PMC4824453; doi:10.1371/journal.ppat.1005481)

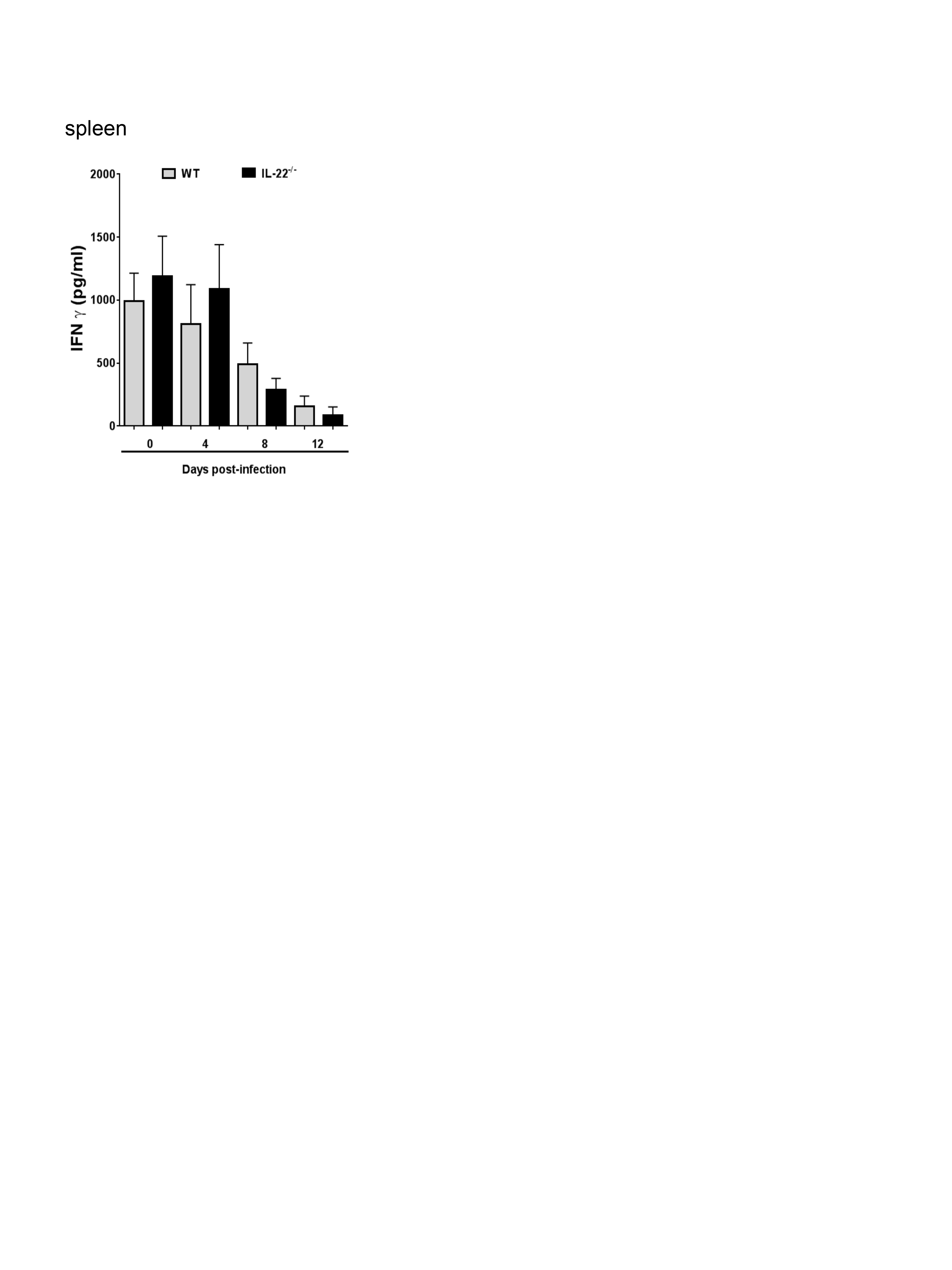

Supplement: S1 Fig — At 4, 8 and 12 days post infection spleen from infected experimental groups were collected, RBCs depleted and cell suspensions generated. Cell suspensions were incubated for 48 hr in presence of conA (5 μg/ml) and supernatants collected. Levels of IFN γ were determined by ELISA as described in methods. Data shown are mean ± SEM from independent experiments where * p<0.05 as compared to strain-matched control and # p<0.05 compared to WT time-matched group (n = 7). (TIF) [file ppat.1005481.s001.tif]

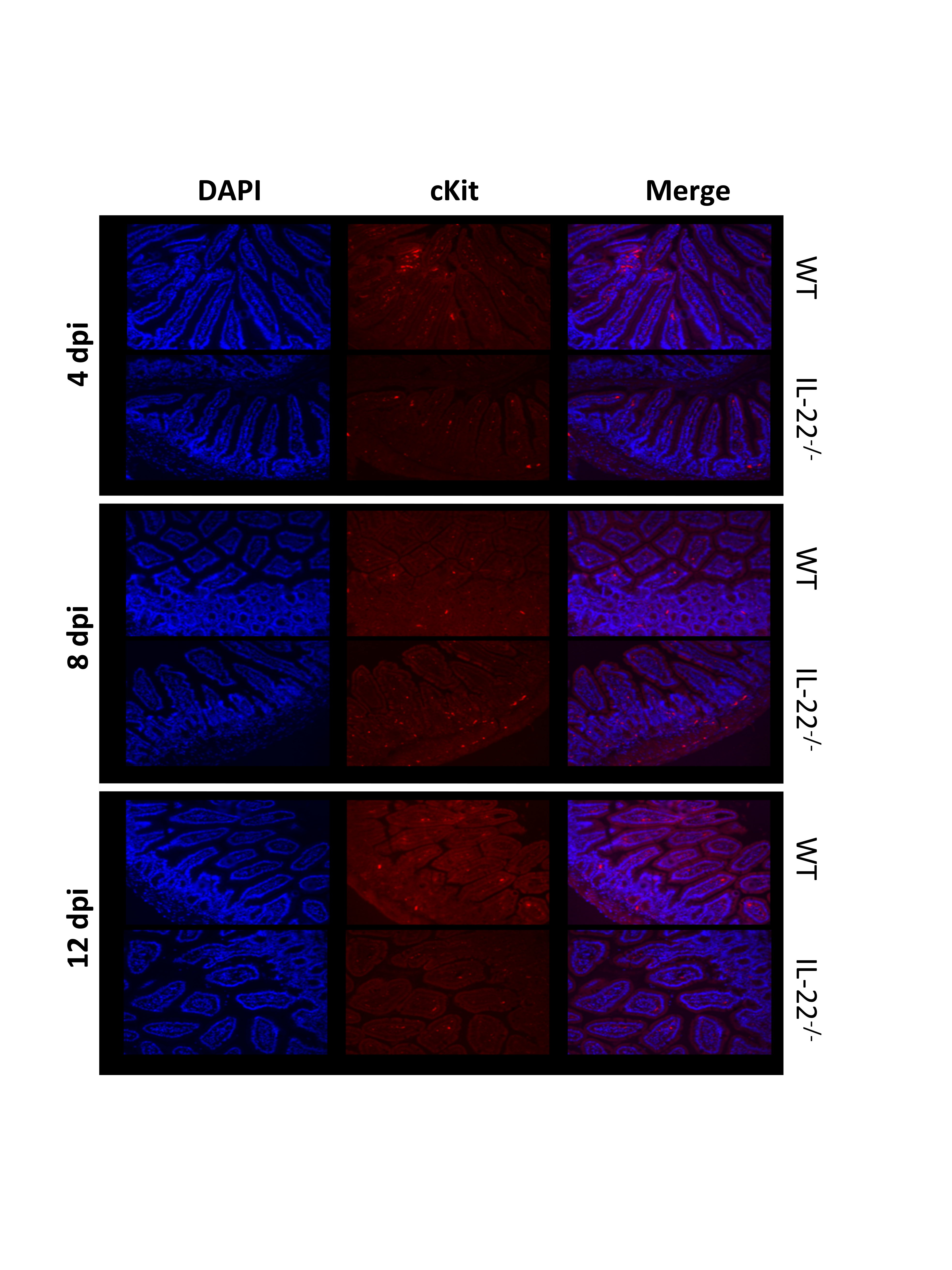

Supplement: S2 Fig — Mice were necropsied at the days post-infection (dpi) indicated, ~1cm of mid-jejunum was collected, fixed, paraffin embedded and immuno-staining performed with anti-cKit antibody (mast cell marker), as per the manufacturer’s instructions, and DAPI staining used to identify nuclei. Random fields of view were chosen based on DAPI staining and observed in a blinded fashion (images are representative of n = 3–4 mice; original mag. = x200). (TIF) [file ppat.1005481.s002.tif]

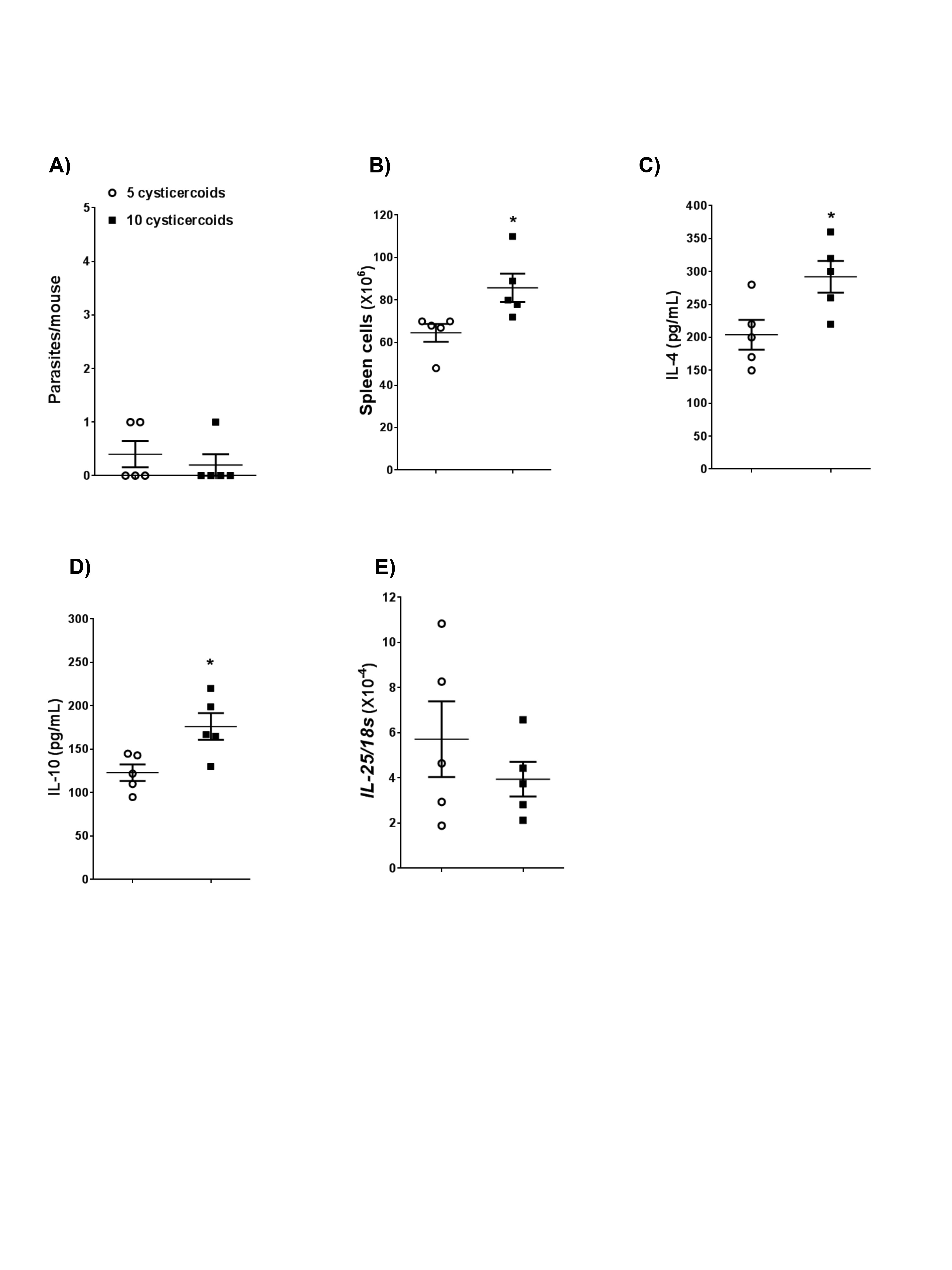

Supplement: S3 Fig — Wild-type mice received 5 or 10 cysticercoids of H. diminuta and on necropsy 8 days later (A) there was no difference in worm expulsion, while (B-D) the number of splenocytes and concanavalin-induced IL-4 and IL-10 production was significantly increased. (E) However, analysis of mid-jejunum segments extracted in Trizol by qPCR revealed no differences in IL-25 mRNA expression. Lines represent mean ± SEM; n = 5; * p<0.05 as compared to animals infected with 5 cysticercoids. (TIF) [file ppat.1005481.s003.tif]

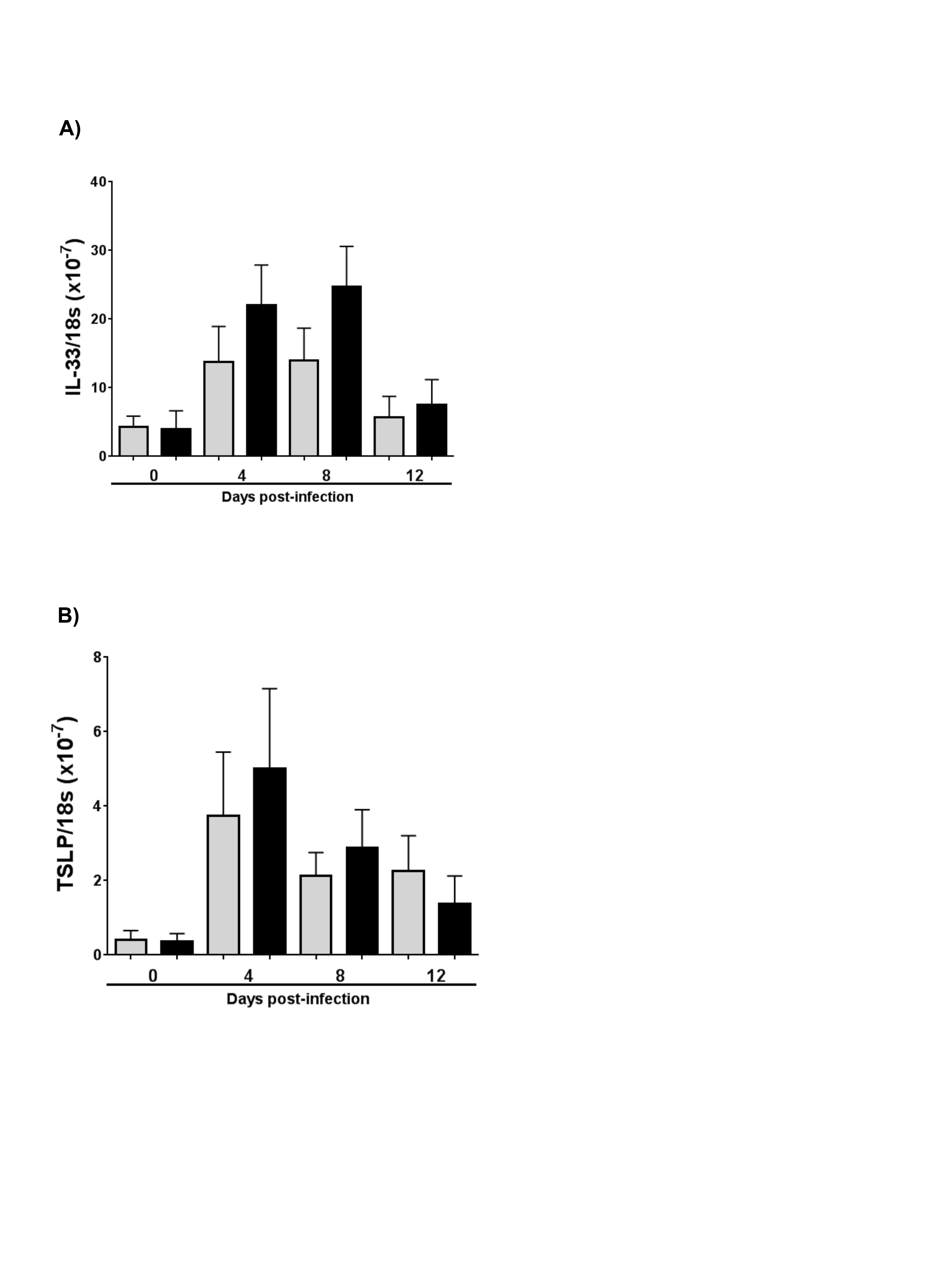

Supplement: S4 Fig — Total mRNA was extracted from small intestine on indicated times after H. diminuta infection from both WT and IL-22-/- mice and (A) IL-33 and (B) TSLP transcripts were measured and normalized against the housekeeping gene 18s. Data shown are mean ± SEM from 2 independent experiments (n- = 6). (TIF) [file ppat.1005481.s004.tif]

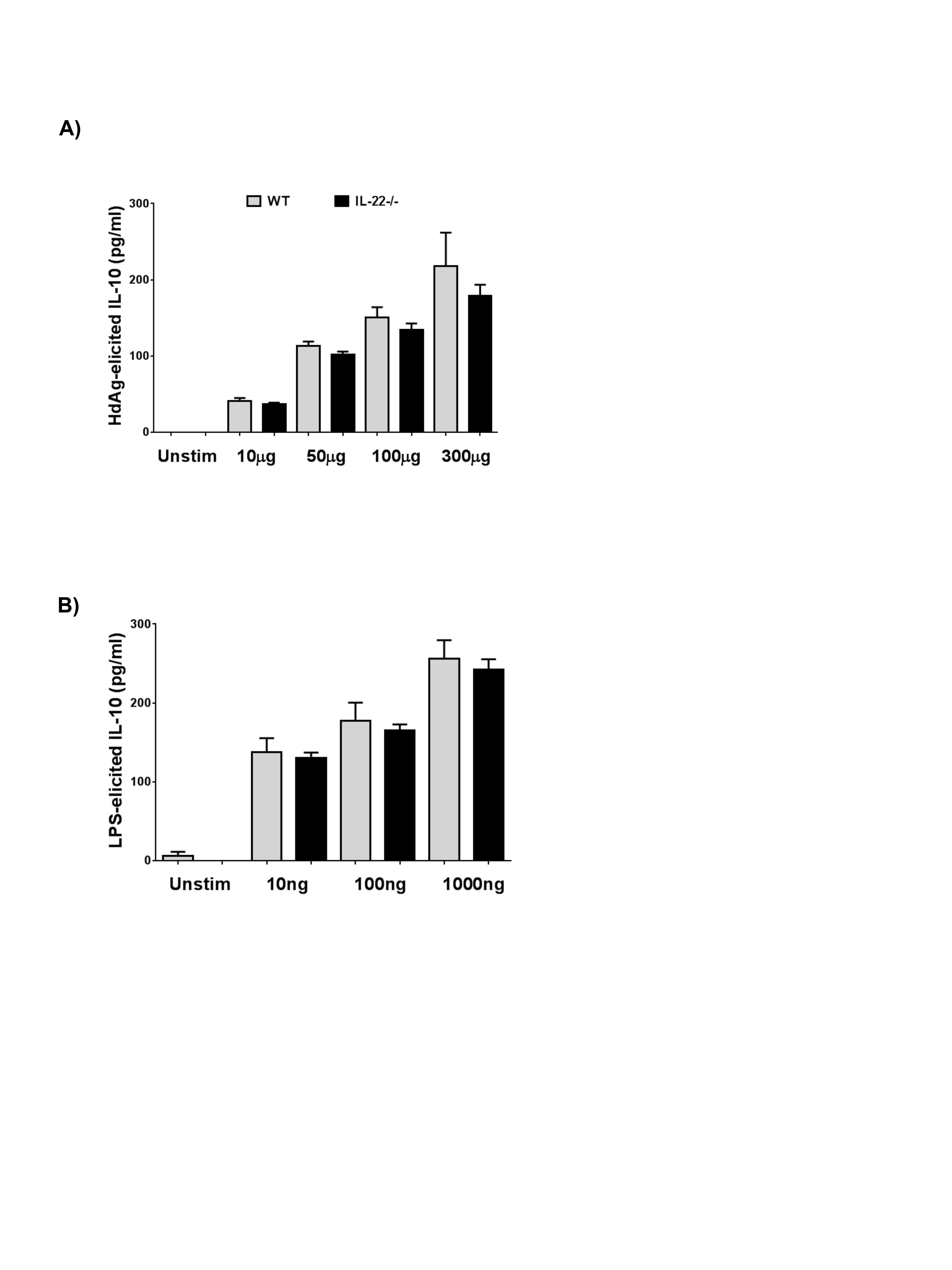

Supplement: S5 Fig — Bone marrow precursors from WT and IL-22-/- mice were differentiated into macrophages for 7 days as described in methods. Upon additional 24 hr of stimulation, supernatans were collected and levels of IL-10 in response to H. diminuta crude antigens (A) and LPS (B) were determined by ELISA. Data shown are from 2 independent experiments with similar results (n = 6). (TIF) [file ppat.1005481.s005.tif]

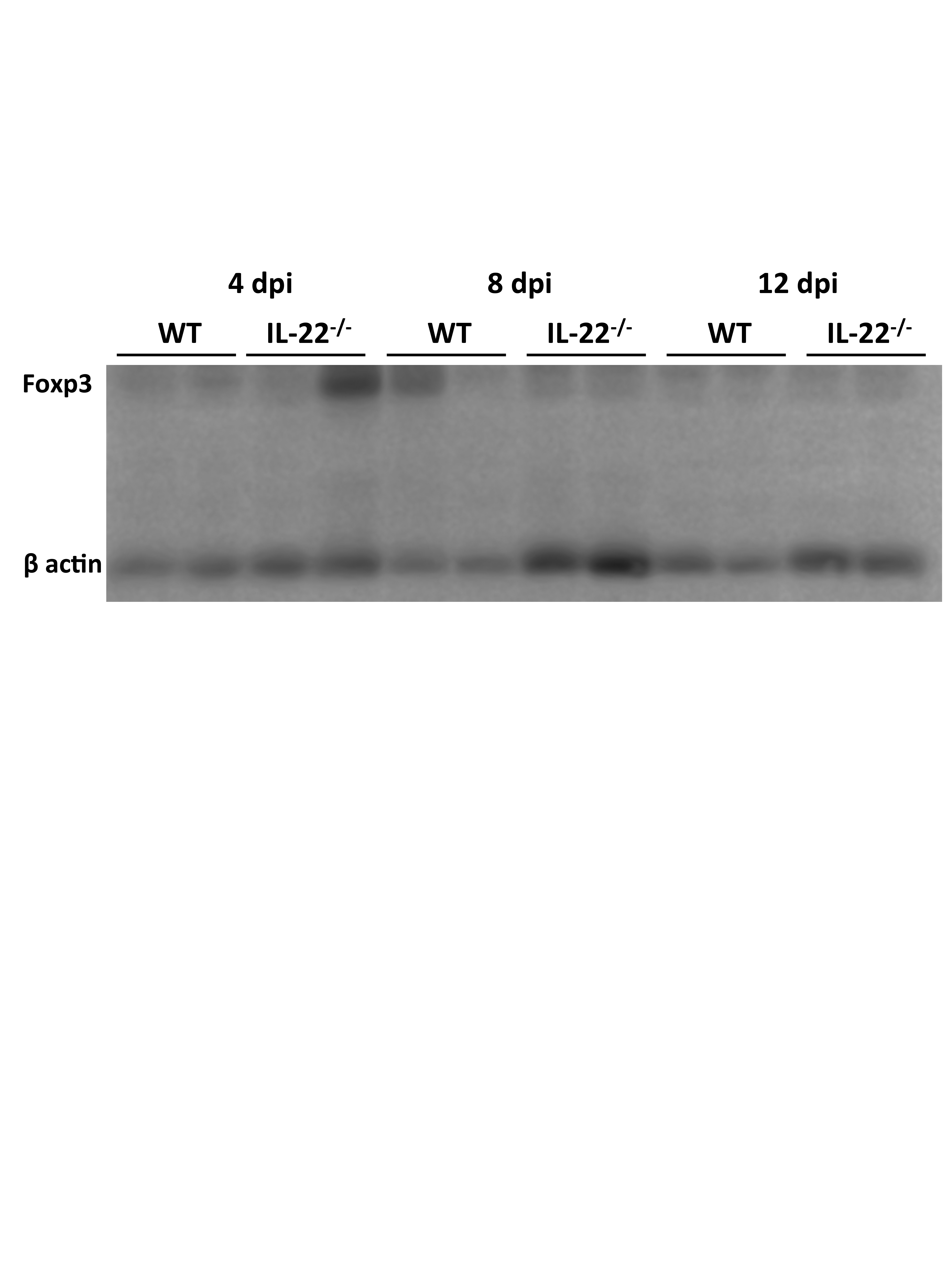

Supplement: S6 Fig — At indicated times post-infection small intestine tissue from both WT and IL-22-/- mice was homogenized in RIPA buffer and total protein extraction was conducted as indicated in methods and Foxp3 protein levels were determined. Beta-actin was used as loading control. Image is representative of 2 experiments with similar results. (TIF) [file ppat.1005481.s006.tif]

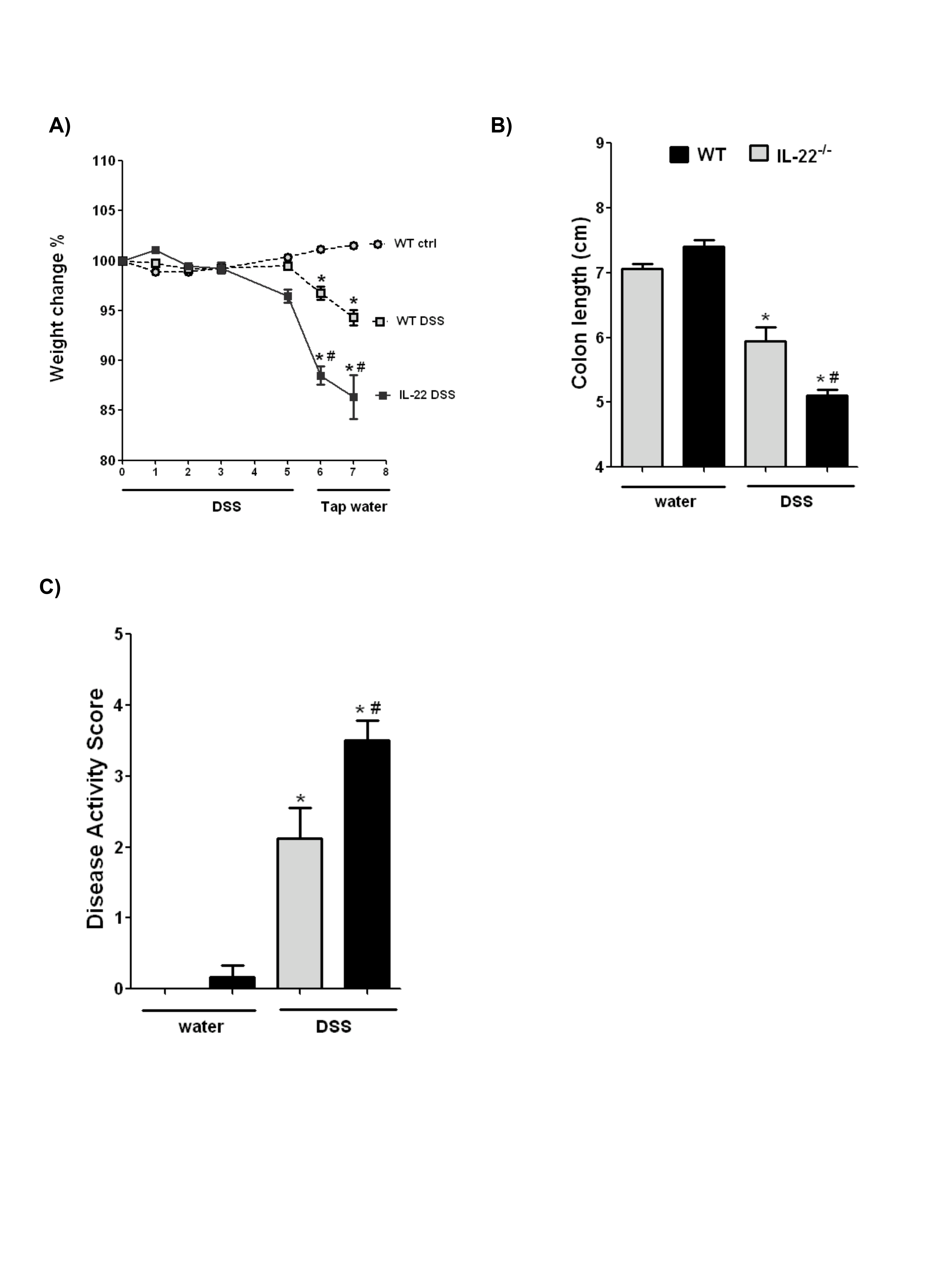

Supplement: S7 Fig — Wild-type (WT) and IL-22-/- mice were exposed to 2.5% (wt./vol.) dextran sodium sulfate (DSS) for 5 days followed by 3 days of normal drinking water and on necropsy IL-22-/- mice displayed increased disease severity as assessed by (A) weight loss, (B) colon length, and (C) disease activity scores (DAS) (data are mean ± SEM; n = 5; * and #, p<0.05 compared to appropriate strain control (ctrl) and WT DSS mice, respectively). (TIF) [file ppat.1005481.s007.tif]

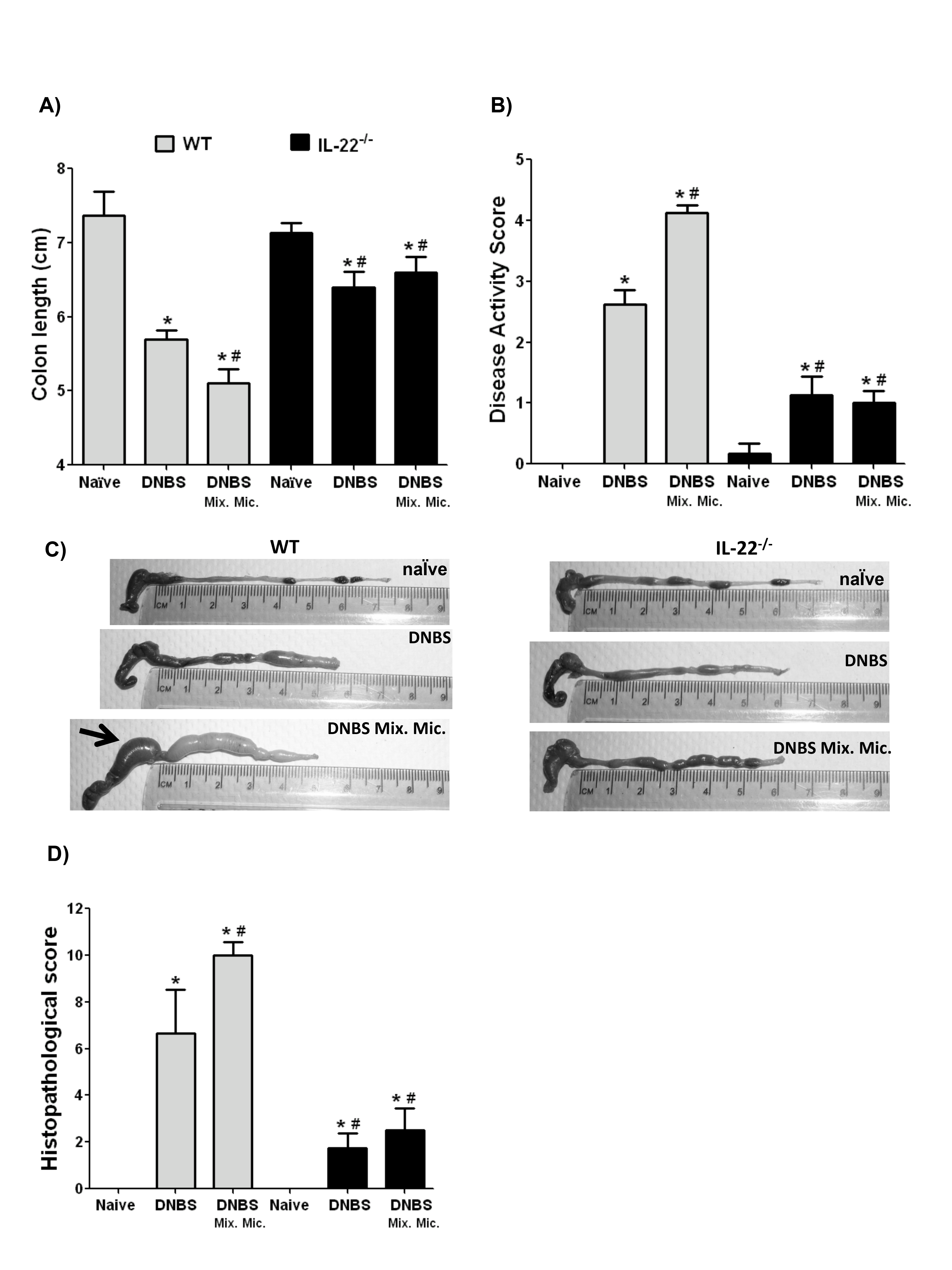

Supplement: S8 Fig — The microbiotas were blended (Mix. Mic.) between wild-type (WT; gray bars) and IL-22-/- mice (black bars) by cross-cage exchange and exploiting the coprophagic behavior of mice, followed by DNBS (5 mg, ir, 72 hr) treatment. The mixed or blended microbiota in IL-22-/- mice did not affect their susceptibility to DNBS, with both groups having less severe colitis than WT mice assessed by colon shortening (A) and overall macroscopic score (B). Representative colon images in (C) show a reduced severity in IL-22-/- mice regardless of having acquired microbiota from WT mice. In contrast, WT mice receiving microbiota from IL-22-/- mice had the most severe colitis. Also, analysis of blind-scored H&E colon sections (D), confirmed less histopathological damage in absence of IL-22. Data are mean ± SEM; n = 5; * and #, p<0.05 compared to the appropriate strain matched control naïve mice and WT DNBS mice, respectively; arrow indicates enlarged caecum. (TIF) [file ppat.1005481.s008.tif]

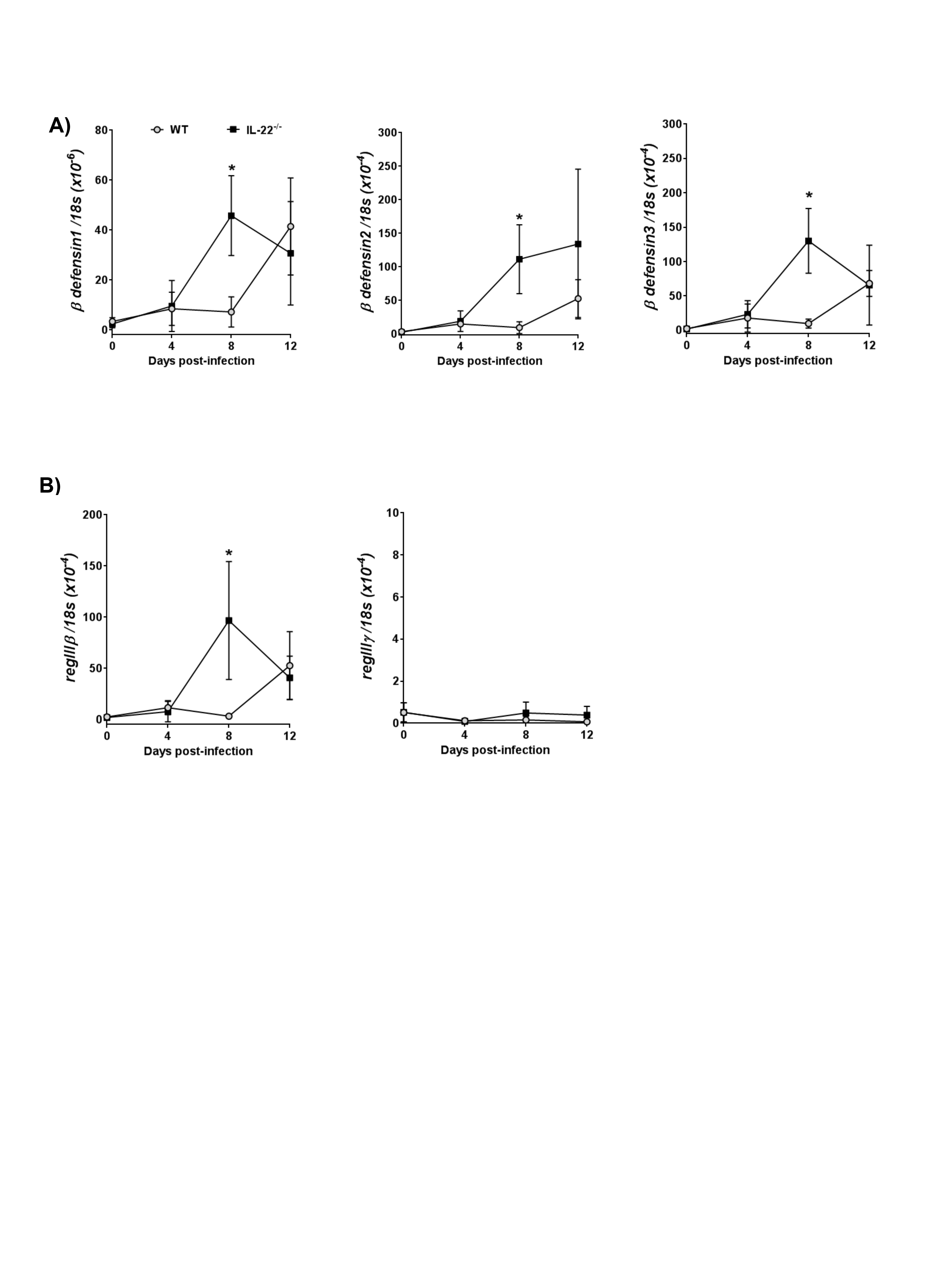

Supplement: S9 Fig — Mid-jejunum tissue was homogenized in Trizol at the indicated time points and mRNA extracted as described in methods. Gene expression of Defensins 1–3 (A) and Reg III beta and gamma peptides was determined by using the specific primers quoted in S10 Fig Data are mean ± SEM from 2 independent experiments (n = 6), p< 0.0.5 as compared to expression found in wild-type (WT) animals. (TIF) [file ppat.1005481.s009.tif]

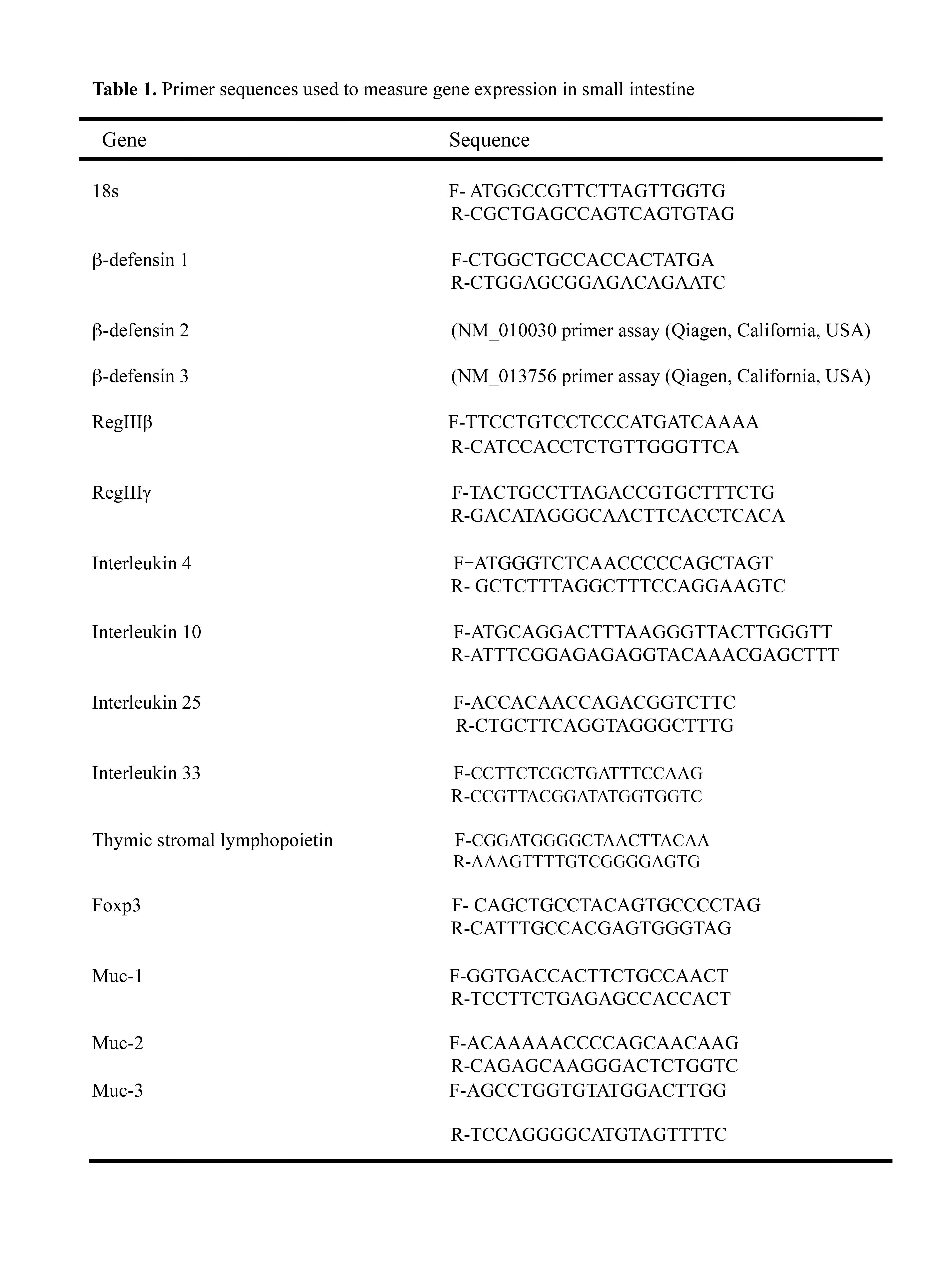

Supplement: S10 Fig — Sequences were syntethized in Univ. of Calgary DNA core facilities or when indicated (i.e. β defensin 2 and β defensin 3) sequences were obtained as ready-to-use primer assay from Qiagen. Muc; mucin, Reg; Regeneration islet-derived protein. (TIF) [file ppat.1005481.s010.tif]
